# Photo‐metallo‐immunotherapy: Fabricating Chromium‐Based Nanocomposites to Enhance CAR‐T Cell Infiltration and Cytotoxicity against Solid Tumors

**DOI:** 10.1002/adma.202407425

**Published:** 2024-07-01

**Authors:** Qingshuang Zou, Ke Liao, Guangchao Li, Xin Huang, Yongwei Zheng, Gun Yang, Min Luo, Evelyn Y. Xue, Chuanqing Lan, Shuai Wang, Yao Shen, Dixian Luo, Dennis K. P. Ng, Quan Liu

**Affiliations:** ^1^ Department of Laboratory Medicine Huazhong University of Science and Technology Union Shenzhen Hospital (Nanshan Hospital) Shenzhen University Shenzhen 518052 China; ^2^ Department of Chemistry The Chinese University of Hong Kong Shatin, N.T. Hong Kong 999077 China; ^3^ Institute of Pharmacy and Pharmacology School of Pharmaceutical Science Hengyang Medical School University of South China Hengyang Hunan 421001 China; ^4^ Department of Hematology The Affiliated Guangdong Second Provincial General Hospital of Jinan University Guangzhou 510317 China; ^5^ Research and Development Department Guangzhou Bio‐Gene Technology Co. Ltd. Guangzhou 510530 China

**Keywords:** cancer immunotherapy, CAR‐T cells, chromium nanoparticles, tertiary lymphoid structure, tumor infiltration

## Abstract

The infiltration and cytotoxicity of chimeric antigen receptor (CAR)‐T cells are crucial for effective elimination of solid tumors. While metallo‐immunotherapy is a promising strategy that can activate the antitumor immunity, its role in promoting CAR‐T cell therapy remains elusive. The first single‐element nanomaterial based on chromium nanoparticles (Cr NPs) for cancer photo‐metallo‐immunotherapy has been reported previously. Herein, an extended study using biodegradable polydopamine as a versatile carrier for these nanoparticles, enabling synergistic CAR‐T cell therapy, is reported. The results show that these nanocomposites with or without further encapsulation of the anticancer drug alpelisib can promote the CAR‐T cell migration and antitumor effect. Upon irradiation with near‐infrared light, they caused mild hyperthermia that can “warm” the “cold” tumor microenvironment (TME). The administration of B7‐H3 CAR‐T cells to NOD severe combined immunodeficiency gamma mice bearing a human hepatoma or PIK3CA‐mutated breast tumor can significantly inhibit the tumor growth after the induction of tumor hyperthermia by the nanocomposites and promote the secretion of serum cytokines, including IL‐2, IFN‐γ, and TNF‐α. The trivalent Cr^3+^ ions, which are the major degradation product of these nanocomposites, can increase the CXCL13 and CCL3 chemokine expressions to generate tertiary lymphoid structures (TLSs) in the tumor tissues, facilitating the CAR‐T cell infiltration.

## Graphical abstract

Chromium nanoparticles (Cr NPs) coated with a layer of biodegradable polydopamine can empower the chimeric antigen receptor (CAR)‐T cell therapy against solid tumors. Upon laser irradiation, the induced hyperthermia can “warm” the “cold” tumor microenvironment (TME) and boost the therapeutic effect of CAR‐T cells. The Cr^3+^ ions released can also promote the CAR‐T cell migration and infiltration for effective inhibition of tumor growth in tumor‐bearing mice.

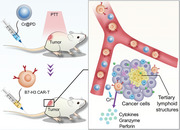

## 1 Introduction

T cells engineered with chimeric antigen receptor (CAR) have led to a remarkable breakthrough in the treatment of hematological malignancies, such as leukemia, non‐Hodgkin B‐cell lymphoma, and myeloma.^[^ ^1^ ^]^ However, the therapeutic efficacy of CAR‐T cells against solid tumors is inconclusive, mostly due to the limited tumor homing and infiltration as well as the inadequate cell killing ability of CAR‐T cells in the immunosuppressive tumor microenvironment (TME).^[^ ^2^ ^]^ To promote tumor homing and direct tumor cell cytolysis by CAR‐T cells, it is important to harness the chemokine system and chemokine expression profile in the TME.^[^ ^3^ ^]^ Evidence has been found that CCL2, CCL3, CCL4, CCL5, and CXCL9 are more highly expressed in melanoma biopsies with increased T‐cell infiltration.^[^ ^4^ ^]^ CAR‐T cells engineered with IL‐7 and CCL19 (7 × 19 CAR) or IL‐7 and CCL21 (7 × 21 CAR) show enhanced infiltration and accumulation in solid tumors, resulting in complete regression of solid tumors and superior antitumor activity.^[^ ^5^ ^]^ In addition, there has been increasing evidence showing that the reconstitution of tertiary lymphoid structures (TLSs) in the TME through the use of key chemokines such as CXCL12 and CXCL13 can increase the CAR‐T cell infiltration and cytotoxicity, leading to improved clinical outcomes.^[^ ^6^ ^]^ For example, the chemokine CXCL13, a transcriptomic marker of TLS, is associated with better patient survival and objective response with immunotherapy involving the formation of TLSs.^[^ ^7^ ^]^ Thus, exploring TLS formation is important for developing new strategies for CAR‐T cell immunotherapy against solid tumors.

Cancer metallo‐immunotherapy can modulate the inherent host and tumor immune signals via metal ions, such as Zn^2+^/Mn^2+^ for amplifying STING activation, Ca^2+^ for T‐cell activation involving lipid regulation, and Fe^2+^, K^+^, Ca^2+^, and Na^+^ for inflammasome activation, to interfere with cancer progression.^[^ ^8^ ^]^ Studies have also shown that Mn^2+^ ions can sensitize the cGAS‐STING pathway, synergizing with cancer phototherapy, chemotherapy, and immunotherapy.^[^ ^9^ ^]^ Moreover, low serum magnesium levels are closely related to fast disease progression and short overall survival during immunotherapy. Magnesium ions can boost CD8+ T‐cell effector and memory functions via LFA‐1 combined with immune checkpoint blockade (ICB) and CAR‐T cell treatments.^[^ ^10^ ^]^ Interestingly, we have recently found that photo‐metallo‐immunotherapy with chromium nanoparticles (Cr NPs), which release Cr^3+^ ions upon degradation, can enhance cancer ICB by promoting the infiltration of macrophages and CD8+ T cells into mouse hepatoma and melanoma via regulating the expression of macrophage inflammatory protein‐1α (MIP‐1α) and the PI3K/Akt/mTOR pathway in macrophages.^[^ ^11^ ^]^ In fact, chromium is an essential nutrient for mammals. The U.S. Food & Drug Administration (FDA)‐approved chromium picolinate is not only a Cr^3+^ supplement but also shows potential in the treatment of insulin resistance, type 2 diabetes, and non‐alcoholic fatty liver disease.^[^ ^12^ ^]^ Despite these promising findings, suitable pharmaceutical forms of Cr NPs that can be used for photo‐metallo‐immunotherapy and loading additional theranostic components have not been worked out, and the use of these nanocomposites to boost CAR‐T cell therapeutic efficacy against solid tumors has not been reported so far.

**Figure 1:**
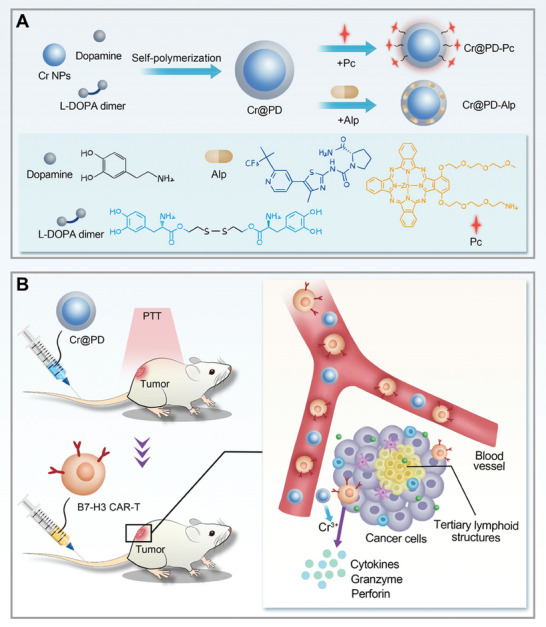
Schematic diagram showing A) the fabrication of various Cr@PD‐based nanosystems and B) their mechanistic action of photo‐metallo‐immunotherapy, promoting the infiltration and cytotoxicity of B7‐H3 CAR‐T cells.

As an extension of our previous study of Cr‐induced photoimmunotherapy,^[^ ^11^ ^]^ we report herein a versatile platform for Cr NPs that can carry additional theranostic agents to extend their function to cancer imaging and enhance their antitumor treatment efficacy. The resulting nanocomposites can also promote the infiltration and cytotoxicity of CAR‐T cells for effective elimination of solid tumors, which remain the major challenges of CAR‐T cell therapy. As shown in **Figure** **1**, the platform contains a coating of biodegradable polydopamine formed by the self‐polymerization of a mixture of dopamine and a glutathione (GSH)‐responsive disulfide‐linked dimer of L‐3,4‐dihydroxyphenylalanine (L‐DOPA) on the Cr NPs. With a layer of adhesive polydopamine, the resulting Cr@PD can conjugate readily with a phthalocyanine‐based fluorescent dye to give Cr@PD‐Pc for tumor imaging.^[^ ^13^ ^]^ It can also encapsulate alpelisib (Alp), a potent chemotherapeutic agent for the treatment of advanced breast cancer harboring the PIK3CA mutation,^[^ ^14^ ^]^ to afford Cr@PD‐Alp that can induce an additional anticancer effect.

In this study, we used CAR‐T cells targeting the antigen B7‐H3 (also known as CD276), which is overexpressed in various cancers, including liver cancer and breast cancer, whereas little or no expression of this antigen is observed in normal tissues.^[^ ^15^ ^]^ Therefore, B7‐H3 is regarded as a promising target for cancer immunotherapy, and previous studies have also indicated that B7‐H3 CAR‐T cell therapy can partially control tumor growth in patients with basal cell carcinoma.^[^ ^16^ ^]^ It was expected that, upon light irradiation, these Cr@PD‐based nanoplatforms can cause local mild hyperthermia to kill cancer cells via photothermal therapy (PTT). The Cr^3+^ ions released from the nanoparticles in the acidic TME can also increase the CAR‐T cell migration as reported previously^[^ ^17^ ^]^ and promote the cell‐killing ability, chemokine secretion, and TLS formation in tissues. Through a series of carefully designed in vitro and in vivo experiments, we have demonstrated that the Cr^3+^ ions released from this versatile platform can elicit robust antitumor immunity by strengthening the cytotoxic effect of B7‐H3 CAR‐T cells, as indicated by the increased expression of perforin and granzyme, the extensive formation of TLSs, and the enhanced tumor infiltration of CAR‐T cells (Figure 1). The overall results show that these Cr‐based nanocomposites can function as novel and highly efficient therapeutics for photo‐metallo‐immunotherapy against solid tumors.

## 2 Results and Discussion

### 2.1 Fabrication, Characterization, and Physical Properties of Cr‐Based Nanoparticles

Nanoscale Cr particles were first prepared by ultrasonic peeling of commercial Cr particles with a dimension of 5–10 µm in deionized water, followed by centrifugation and filtration to collect the NPs with a size of less than 0.1 µm. These Cr NPs were then dispersed in a mixture of dopamine and the previously reported disulfide‐linked L‐DOPA dimer^[^ ^13^ ^]^ in a 10 mM Tris HCl buffer at pH 8.5. Upon irradiation in an ultrasonic bath at room temperature for 3 h, dopamine and the L‐DOPA dimer underwent self‐polymerization to form a dense layer of mixed polydopamine on the Cr NPs, resulting in the formation of the nanocomposite Cr@PD. The introduction of the GSH‐cleavable L‐DOPA dimer enables the polydopamine layer to be degraded by the intracellular GSH to facilitate the release of Cr^3+^ ions. As expected, the size of these NPs depended on the ratio of Cr NPs, dopamine, and the L‐DOPA dimer used. As shown in Table S1 (Supporting Information), the hydrodynamic diameter as determined by dynamic light scattering (DLS) ranged from 68 to 370 nm for the various ratios we used. The NPs prepared by using a ratio of 1:0.5:0.5 (by weight) with a hydrodynamic diameter of 83.5 ± 18.3 nm were used for further fabrication.

To incorporate an anticancer agent into the NPs, the new targeted drug Alp for advanced breast cancer harboring the PIK3CA mutation was also loaded during the self‐polymerization process to give Cr@PD‐Alp, in which molecules of Alp were encapsulated in the polydopamine layer. To facilitate the imaging of the NPs during the cell internalization, a phthalocyanine‐based fluorescent probe was also immobilized on the surface of Cr@PD. It was achieved by simply stirring a mixture of these NPs and our previously reported amino phthalocyanine Pc^[^ ^18^ ^]^ in water containing 0.5% Tween 20 at room temperature for 16 h. Through Schiff base reaction or Michael addition, molecules of Pc could be conjugated to the surface of Cr@PD‐Pc. The loading of Alp and Pc was determined by electronic absorption spectroscopy to be 16.4 µg mL^−1^ and 3.5 µM, respectively, in 100 µg mL^−1^ Cr@PD, giving a loading efficiency of 82% and 35%, respectively.

**Figure 2:**
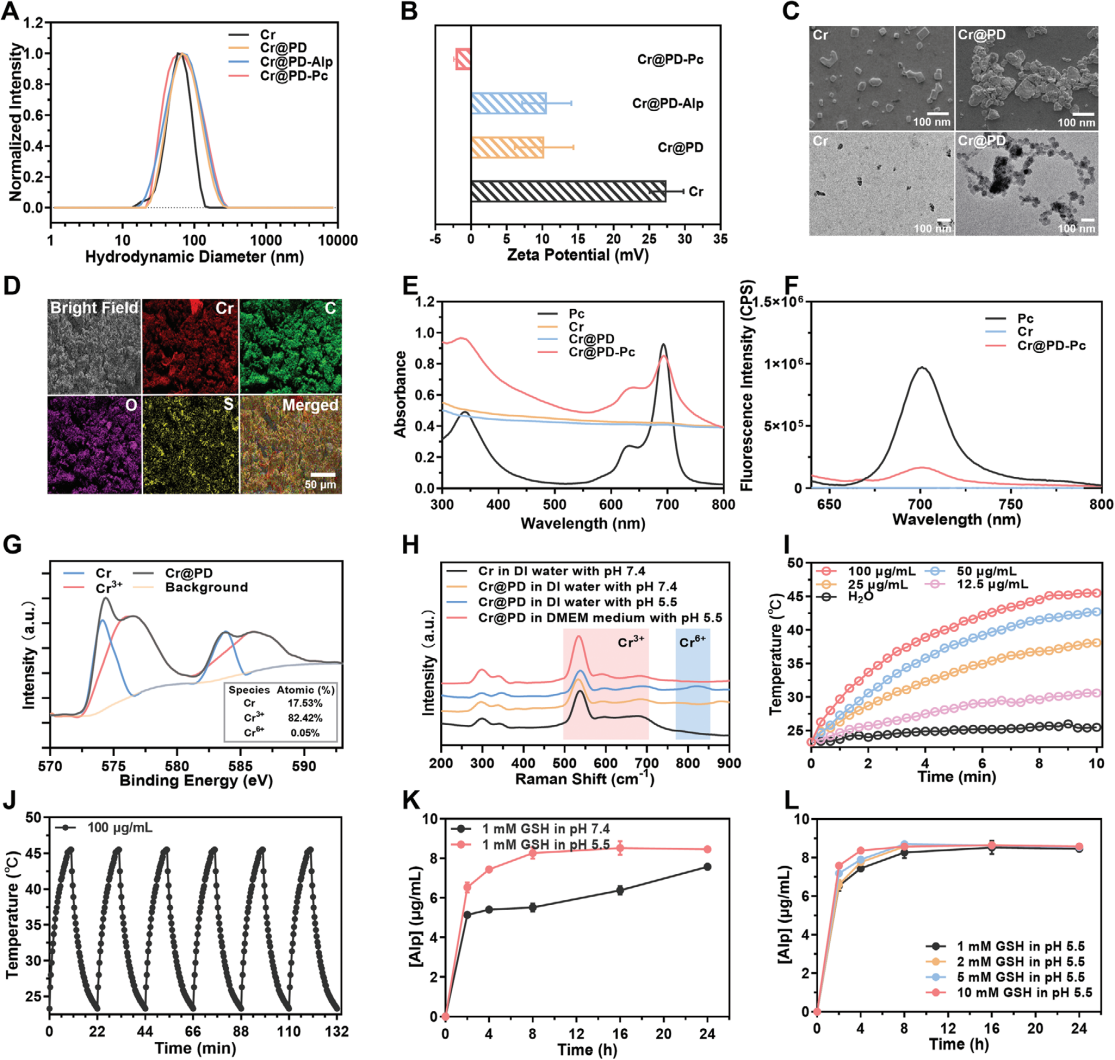
A) Normalized hydrodynamic diameters and B) zeta potentials of different Cr‐based NPs. C) Scanning (top) and transmission (bottom) electron microscopic images of Cr and Cr@PD NPs. D) EDS results for Cr@PD. E) Electronic absorption and F) fluorescence (λ_ex_ = 610 nm) spectra of free Pc (at 3.5 µM with 0.5% Tween 20) and different Cr‐based NPs in deionized water (at 100 µg mL^−1^). G) XPS results for Cr@PD in deionized water after being left under ambient conditions for two weeks. H) Raman spectra of Cr and Cr@PD NPs in different media recorded after putting the dispersions aside under ambient conditions for two weeks. I) Photothermal effect of Cr@PD in deionized water upon irradiation with an 808 nm laser operated at 1 W cm^−2^. J) Temperature cycling curve for Cr@PD in deionized water (at 100 µg mL^−1^). Each cycle involves laser irradiation for 10 min followed by natural cooling for 12 min. K) Release profiles of Alp upon treatment of Cr@PD‐Alp (at 100 µg mL^−1^) with GSH (1 mM) in deionized water at pH 7.4 and 5.5. L) Release profiles of Alp upon treatment of Cr@PD‐Alp (at 100 µg mL^−1^) with different concentrations of GSH (1–10 mM) in deionized water at pH 5.5. For B), K), and L), data are expressed as the mean ± standard deviation (SD) of three independent measurements.

All these nanocomposites were first characterized with DLS. As expected, the coating of a polydopamine layer on the Cr NPs increased the hydrodynamic diameter (from 68.2 ± 4.2 to 83.5 ± 18.3 nm). After the incorporation of Alp and Pc, the hydrodynamic diameter was slightly increased to 92.3 ± 12.2 and 86.6 ± 15.6 nm, respectively (**Figure** **2**A). The zeta potential of Cr NPs was remarkably changed upon the surface modifications as shown in Figure 2B. The morphology of Cr@PD was also examined by scanning electron microscopy and transmission electron microscopy (TEM), which showed that the NPs tend to aggregate upon coating with polydopamine (Figure 2C). Energy‐dispersive X‐ray spectroscopy (EDS) further revealed the presence of chromium, carbon, oxygen, and sulfur in Cr@PD (Figure 2D), which was consistent with the presence of a polydopamine layer formed by self‐polymerization of dopamine and the disulfur‐linked L‐DOPA dimer coated on the Cr NPs. For Cr@PD‐Pc, the presence of Pc was confirmed by the characteristic Q‐band absorption of zinc(II) phthalocyanines at ≈680 nm in the absorption spectrum (Figure 2E). Its fluorescence band (at ≈710 nm) was significantly diminished due to self‐quenching as observed previously for Pc‐immobilized polydopamine NPs (Figure 2F).^[^ ^13^ ^]^

To reveal the fate of Cr in the NPs, X‐ray photoelectron spectroscopy (XPS) was used to examine the dispersion of Cr@PD in deionized water after being left under ambient conditions for two weeks. As shown in Figure 2G, most of the Cr existed as the trivalent Cr^3+^ ions (82.42%) with a small fraction remained as Cr(0) (17.53%) and negligible Cr^6+^ ions (0.05%), showing that the NPs underwent extensive degradation during this period of time to release Cr^3+^ ions. Similar results were obtained by using Raman spectroscopy to monitor the degradation of Cr and Cr@PD NPs in different media. As shown in Figure 2H, for the dispersions of Cr (at pH 7.4) and Cr@DP (at pH 7.4 and 5.5) NPs in deionized water and that of Cr@DP in Dulbecco's Modified Eagle Medium (DMEM) at pH 5.5, all being left under ambient conditions for two weeks, the characteristic bands for Cr(0) and Cr^3+^ at 300–350 and 535–665 cm^−1^, respectively, were observed, while the characteristic band for Cr^6+^ at 780–825 cm^−1^ was not noticeable, which supported that the trivalent Cr^3+^ ions were the major degradation product.^[^ ^11^ ^]^

With a Cr nanocore coated with polydopamine, Cr@PD was expected to exhibit a photothermal effect. Figure 2I shows the temperature increase curves for different concentrations of Cr@PD in deionized water upon irradiation with an 808 nm laser operated at 1 W cm^−2^. The heating effect was generally increased with the concentration of Cr@PD. At a concentration of 100 µg mL^−1^, the temperature increased to 46 °C after irradiation for 10 min. The response was just slightly stronger than that of Cr NPs (Figure S1, Supporting Information), showing that the photothermal effect was mainly caused by the Cr nanocore instead of the polydopamine layer. The relatively weak photothermal effect of polydopamine has been reported previously, which has been attributed to its incomplete nonradiative transition and high aggregation tendency that will induce weak near‐infrared absorbability and decrease the light absorption efficiency, respectively.^[^ ^19^ ^]^ By using the methodology reported previously,^[^ ^20^ ^]^ the photothermal conversion efficiency of Cr@PD and Cr NPs was determined to be 36.5% and 33.9%, respectively. For Cr@PD, after stopping the irradiation for 12 min, the temperature was dropped to the initial value. After repeating these heating and cooling processes for six cycles, there was no significant decrease in the photothermal efficiency (Figure 2J), indicating that this nanocomposite has excellent photothermal properties.

With disulfide linkages, the polydopamine‐coated NPs were expected to be responsive toward GSH. In fact, for Cr@PD‐Alp in deionized water, the addition of GSH (1 mM) triggered the release of Alp through the degradation of the NPs, as monitored by electronic absorption spectroscopy, and by lowering the pH of the dispersion from 7.4 to 5.5, the release rate was significantly faster (Figure 2K). As shown in Figure 2L, the release rate (for the first 4 h) was also slightly increased when a higher concentration of GSH was used.

All these results showed that the Cr@PD‐based NPs had been fabricated successfully. They underwent degradation gradually to release Cr^3+^ ions and Alp (for Cr@PD‐Alp), and the degradation was promoted by GSH through cleavage of the disulfide bonds in the polydopamine coating, particularly in an acidic medium. These NPs also exhibited excellent photothermal properties, rendering them as promising photothermal agents for elimination of cancer cells and tumors.

### 2.2 Cytotoxicity of Cr@PD‐Based Nanocomposites

**Figure 3:**
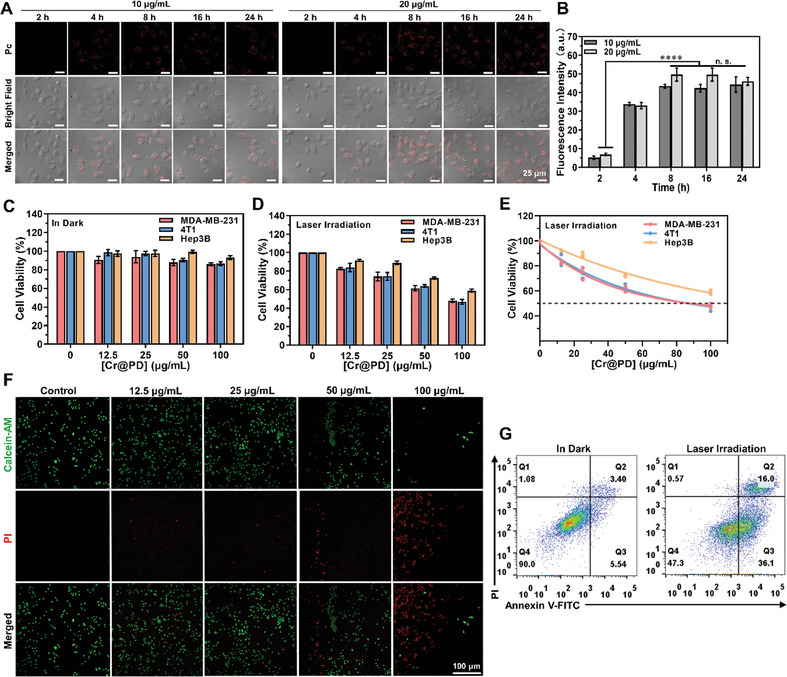
A) Confocal images of MDA‐MB‐231 cells after incubation with Cr@PD‐Pc (10 or 20 µg mL^−1^, which are equiv. to 0.35 and 0.70 µM of Pc, respectively) in RPMI 1640 medium for different periods of time. Scale bar = 25 µm. B) Corresponding quantified intracellular fluorescence intensities. Data are expressed as the mean ± standard error of the mean (SEM) of three independent experiments (n = 50 cells). n.s., not significant; ****p ≤ 0.0001. Concentration‐dependent cytotoxicity of Cr@PD against MDA‐MB‐231, 4T1, and Hep3B cells after incubation for 8 h C) without and D) with further irradiation with an 808 nm laser operated at 1 W cm^−2^ for 10 min. Data are expressed as the mean ± SEM of three independent experiments, each performed in quadruplicate. E) Plot of cell viability against concentration of Cr@PD used for the laser irradiated cells. F) Confocal fluorescence images of MDA‐MB‐231 cells after incubation with different concentrations of Cr@PD for 8 h, laser irradiation (808 nm, 1 W cm^−2^) for 10 min, and then staining with calcein‐AM and PI. Scale bar = 100 µm. G) Flow cytometric analysis of MDA‐MB‐231 cells after incubation with Cr@PD (100 µg mL^−1^) for 8 h, laser irradiation (808 nm, 1  W cm^−2^) for 10 min, and then staining with annexin V‐FITC and PI.

The in vitro properties of these Cr@PD‐based NPs were then studied using several cell lines. To visualize the internalization process, Cr@PD‐Pc was first used for incubation against MDA‐MB‐231 human breast adenocarcinoma cells. **Figure** **3**A shows the confocal images of the cells being incubated with Cr@PD‐Pc (10 or 20 µg mL^−1^) in Roswell Park Memorial Institute (RPMI) 1640 medium for different periods of time (from 2 to 24 h). The corresponding fluorescence intensities were then determined and are depicted in Figure 3B. The results showed that an incubation with 10 µg mL^−1^ for 8 h was sufficient to attain optimal cellular uptake as shown by the almost maximal intracellular fluorescence intensity. These incubation conditions were then used as a reference for the cytotoxicity study.

Apart from this cell line, 4T1 murine mammary cancer cells and Hep3B human hapatocellular carcinoma cells were also used for this study. The cells were incubated with different concentrations of Cr@PD (up to 100 µg mL^−1^) for 8 h with or without further laser irradiation at 808 nm (1 W cm^−2^) for 10 min. The cell viabilities under these conditions were then determined by a cell counting kit 8 (CCK8) assay. As shown in Figure 3C, the cytotoxicity of Cr@PD was negligible for all the three cell lines even at the highest concentration used (100 µg mL^−1^). However, upon laser irradiation, the cell viability was generally decreased with the concentration of Cr@PD (Figure 3D), showing that the NPs exhibited a mild photothermal cell‐killing effect. The half‐maximal inhibitory concentration (IC_50_ value) could barely be determined for MDA‐MB‐231 (91 µg mL^−1^) and 4T1 (88  µg mL^−1^) cells (Figure 3E). At the maximum concentration of Cr@PD used, only about half of the cells were killed. As PTT would mainly be used to promote the efficacy of CAR‐T cell therapy, no attempts were made to further enhance the photocytotoxicity of Cr@PD.

The Cr@PD‐induced cell death of MDA‐MB‐231 cells was further examined using a live/dead double staining protocol based on calcein acetoxymethyl ester (calcein AM) and propidium iodide (PI). As shown in Figure 3F, as the concentration of Cr@PD increased, the green fluorescence due to calcein‐AM (which indicates the live cells) became weaker and the red fluorescence due to PI (which indicates the dead cells) was intensified, showing that more and more cells were killed when a higher concentration of Cr@PD was used. By using the double staining assay with annexin V – fluorescein isothiocyanate (FITC) and PI to examine the cells after the treatment with 100 µg mL^−1^ of Cr@D, followed by laser irradiation (808 nm, 1 W cm^−2^) for 10 min, it was found that ≈52% of the cancer cells were either in the early (Q3) or late (Q2) apoptotic stage (Figure 3G).

### 2.3 PTT‐Promoted Cytotoxicity of Migrated CAR‐T Cells

**Figure 4:**
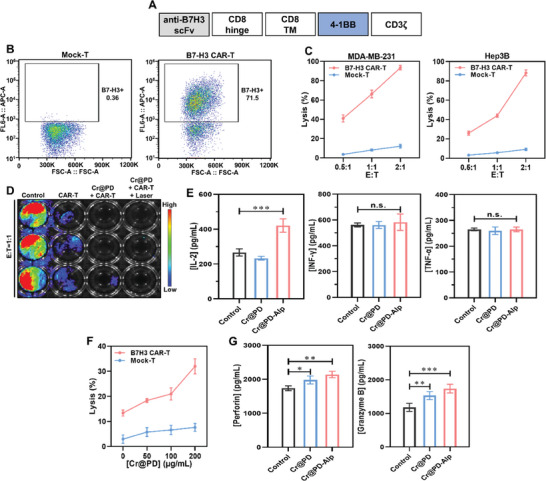
A) Schematic diagram of the B7‐H3 CAR‐T vector. B) Percentages of scFv expression in Mock‐T and B7‐H3 CAR‐T cells determined by flow cytometry. C) Cell‐killing effect of Mock‐T and B7‐H3 CAR‐T cells on MDA‐MB‐231 and Hep3B cells after coculturing for 24 h at different E:T ratios. D) Cell‐killing effect of B7‐H3 CAR‐T cells against Hep3B cells after coculturing for 24 h at an E:T ratio of 1:1 with or without further treatment with Cr@PD (200 µg mL^−1^) for 24 h and laser irradiation (808 nm, 1 W cm^−2^) for 10 min as shown by bioluminescence imaging. E) IL‐2, IFN‐γ, and TNF‐α concentrations in the cell supernatants measured by ELISA kits after incubating B7‐H3 CAR‐T cells with Cr@PD or Cr@PD‐Alp (200 µg mL^−1^) for 24 h. F) Cytotoxicity of migrated B7‐H3 CAR‐T cells against Hep3B cells in Transwell plates after incubation with different concentrations of Cr@PD for 6 h. G) Levels of granzyme and perforin B released from B7‐H3 CAR‐T cells triggered by Cr@PD and Cr@PD‐Alp (200 µg mL^−1^). For C) and E‐G), data are expressed as the mean ± SD of three independent measurements. *p < 0.05, **p < 0.01, and ***p < 0.001.

B7‐H3 CAR‐T cells were then designed to target B7‐H3‐expressing tumors. According to the procedure reported previously,^[^ ^16^ ^]^ a humanized single‐chain variable fragment (scFv) targeting B7‐H3 was utilized to construct the CAR sequence, which together with the hinge region of mCD8, the transmembrane and costimulatory regions of CD8, and the CD3 ζ‐signaling region formed B7‐H3 CAR‐T cells (**Figure** **4**A). The expression of B7‐H3 scFv in the T cells was determined to be 71.5% using flow cytometry (Figure 4B). The specific cell‐killing effect of B7‐H3 CAR‐T cells was demonstrated using MDA‐MB‐231 and Hep3B cells. After coculturing for 24 h, the B7‐H3 CAR‐T cells generally exhibited much stronger cell‐killing ability than the Mock‐T cells at different effector‐to‐target (E:T) ratios (Figure 4C). In addition, we also cocultured B7‐H3 CAR‐T and Hep3B cells at an E:T ratio of 1:1 and further determined the cell‐killing ability of B7‐H3 CAR‐T cells with the treatment of Cr@PD (200 µg mL^−1^) for 24 h and laser irradiation (808 nm, 1 W cm^−2^) for 10 min. It was found that Cr@PD and B7‐H3 CAR‐T cells exerted a significant synergistic effect in killing the cancer cells, and the cytotoxicity was further increased upon laser irradiation (Figure 4D).

To further investigate the effects of Cr@PD and Cr@PD‐Alp on the cell‐killing ability of B7‐H3 CAR‐T cells, we measured the cytokine levels in the cell supernatant by using ELISA kits after incubating B7‐H3 CAR‐T cells with Cr@PD or Cr@PD‐Alp for 24 h. The results showed that, with the exception of IL‐2 expression by Cr@PD‐Alp, both nanocomposites did not significantly change the expression of IL‐2, IFN‐γ, and TNF‐α in B7‐H3 CAR‐T cells (Figure 4E). In our previous study, we found that Cr^3+^ ions could promote the migration of macrophages and T lymphocytes.^[^ ^11^ ^]^ Therefore, we examined the cytotoxicity of migrated B7‐H3 CAR‐T cells against Hep3B cells in Transwell plates. After incubation with an increasing concentration of Cr@PD for 6 h, we found that the B7‐H3 CAR‐T cells became more effective in killing the target Hep3B cells, and the efficiency was much higher than that of the Mock‐T cells (Figure 4F). To rationalize the enhancement, 5 × 10^5^ Hep3B cells were seeded in the lower chamber, and 1 × 10^6^ B7‐H3 CAR‐T cells were added to the upper chamber after 12 h. We then added Cr@PD or Cr@PD‐Alp (200 µg mL^−1^) to the lower chamber and collected the cell supernatant from the lower chamber after 6 h. The levels of granzyme and perforin B in the cell supernatant were then measured by using ELISA kits after 24 h. The results showed that both Cr@PD and Cr@PD‐Alp could promote the secretion of perforin and granzyme B (Figure 4G) from B7‐H3 CAR‐T cells, thereby enhancing the cell‐killing effect.

### 2.4 Cr@PD‐Enhanced In Vivo Antitumor Efficacy of B7‐H3 CAR‐T Cells

The in vivo biodistribution of these NPs was first studied using Cr@PD‐Pc, which was incorporated with Pc as a fluorescent probe. BALB/c nude mice bearing an MDA‐MB‐231 tumor were intravenously injected with either phosphate‐buffered saline (PBS) or Cr@PD‐Pc in PBS (1 mg mL^−1^, 200 µL). The fluorescence images of the mice at 24 and 48 h post‐injection were then captured with an imaging system. The tumor and the major organs, including heart, liver, spleen, lung, and kidneys were also harvested at these time points and imaged. It was found that the fluorescence intensity remained strong in the bodies of the mice at 24 h post‐injection, but it was significantly reduced at 48 h post‐injection. However, the fluorescence intensities at the tumor and the organs were not significantly changed (Figure S2, Supporting Information). The high intensity at the tumor showed that the NPs could accumulate in the tumor and retain therein for at least 48 h probably due to the enhanced permeability and retention effect.^[^ ^21^ ^]^

Since the NPs were also significantly accumulated in the liver and kidneys as shown in Figure S2B (Supporting Information), we conducted a detailed study of the toxicity of Cr@PD to C57BL/6 mice. After intravenous administration (10 mg kg^−1^, 100 µL), blood samples were collected on days 1, 7, and 30. Standard blood biochemical indexes for liver and kidney functions, including the total protein, albumin, aspartate aminotransferase, alanine aminotransferase, creatine, urea, and uric acid were determined and compared with those of the control, which involved intravenous injection of PBS only. The results showed no significant abnormalities in both groups of mice (Figure S3, Supporting Information). In addition, hematoxylin and eosin (H&E) staining of the major organs of the mice, including heart, liver, spleen, lung, kidney, brain, and intestine did not reveal any significant acute or chronic pathologic toxicity on days 1, 7, and 30 after the injection (Figure S4, Supporting Information). To evaluate the long‐term toxicity of this nanosystem, Cr@PD in PBS, both at a dose of 10 mg kg^−1^ and 20 mg kg^−1^ (100 µL), was injected intravenously to C57BL/6 mice, and similar blood and pathological tests were conducted after 90 days, using PBS‐treated mice for comparison. As shown in Figure S5 (Supporting Information), all the results of complete blood count and biochemical tests of the liver and kidney functions as well as the pathological changes of the major organ tissues did not differ significantly. Even by increasing the drug dose by twofold, the toxicity was not noticeable. These results indicated that Cr@PD exhibited good biocompatibility and could be safely used for further in vivo experiments.

**Figure 5:**
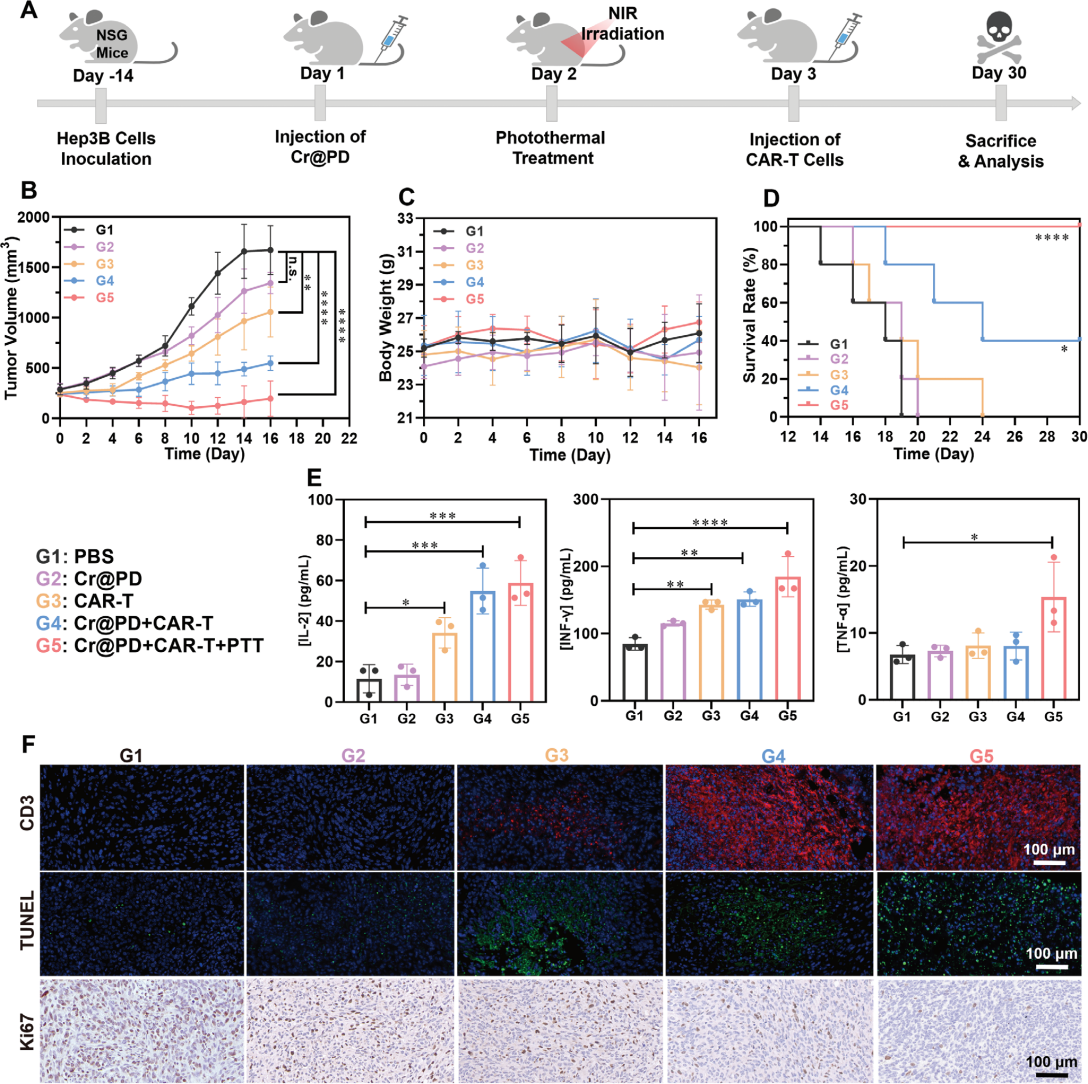
A) Timeline of the in vivo study of the antitumor efficacy of Cr@PD and B7‐H3 CAR‐T cells against Hep3B tumor‐bearing NSG mice. B) Tumor growth curves for the mice receiving different treatments: (G1) intravenous injection with PBS (100 µL); (G2) intravenous injection with Cr@PD in PBS (10 mg kg^−1^, 100 µL); (G3) intravenous injection with B7‐H3 CAR‐T cells (3 × 10^6^, 100 µL); (G4) intravenous injection with Cr@PD in PBS (10 mg kg^−1^, 100 µL) followed by intravenous injection with B7‐H3 CAR‐T cells (3 × 10^6^, 100 µL) after 48 h; (G5) intravenous injection with Cr@PD in PBS (10 mg kg^−1^, 100 µL) followed by laser irradiation (808 nm, 1 W cm^−2^, 8 min) at the tumor site after 24 h, and then intravenous injection with B7‐H3 CAR‐T cells (3 × 10^6^, 100 µL) after a further 24 h (n = 5). C) Changes in the body weights of the mice in different groups (n = 5). D) Kaplan‒Meier survival curves of the mice in different groups (n = 5). E) Quantitative analysis of serum cytokines IL‐2, IFN‐γ, and TNF‐α for the mice in different groups on day 30 by ELISA (n = 3). F) Immunofluorescence staining for CD3, TUNEL staining, and immunohistochemical staining for Ki67 in tumors after the above treatments. Scale bar = 100 µm. For B) to E), data are expressed as the mean ± SD (n = 5 or 3). *p < 0.05, **p < 0.01, ***p < 0.001, and ****p < 0.0001.

To investigate the effect of the photothermal response using Cr@PD on the antitumor efficacy of B7‐H3 CAR‐T cells, we established a subcutaneous Hep3B liver tumor model in which Hep3B cells were mixed with stromal gel in NOD severe combined immunodeficiency (SCID) gamma (or simply NSG) mice. After 14 days, the mice were randomly divided into five groups (n = 5). For the positive treatment group (group five), the mice were injected intravenously with Cr@PD in PBS (10 mg kg^−1^, 100 µL). After 24 h, the tumor site was irradiated with an 808 nm laser (1 W cm^−2^) for 8 min to induce PTT. After a further 24 h, B7‐H3 CAR‐T cells were injected intravenously into the mice as shown in **Figure** **5**A. For comparison, the mice were simply treated with PBS (group one) or Cr@PD (group two) without further treatments, with B7‐H3 CAR‐T cells only (group three), or with Cr@PD and B7‐H3 CAR‐T cells without laser irradiation (group four). The corresponding tumor‐grow curves are shown in Figure 5B. It can be seen that the tumor‐inhibition efficacy follows the order: G1 < G2 < G3 < G4 < G5, and for treatment group five, the tumors could be completely eliminated. These results showed that the mice, after being treated with PTT with Cr@PD, had a significantly improved antitumor response toward B7‐H3 CAR‐T cells. For all the above treatments, the body weight of the mice did not significantly change over 16 days (Figure 5C). In addition, according to the results of Kaplan‒Meier survival analysis, the survival of NSG mice in groups four and five was prolonged compared with that in the other three control groups. For group five, all the mice remained survived after 30 days (Figure 5D). It is likely that the mild photothermia induced by Cr@PD triggered the immunostimulatory effect and promoted the endogenous immune cell recruitment and infiltration, which further enhanced the antitumor effect of CAR‐T cells. To provide further evidence, the serum cytokine levels of the mice in each group at the end of the treatments, i.e., day 30, were measured using ELISA kits. It was found that the IL‐2, IFN‐γ, and TNF‐α levels were significantly elevated for groups three to five compared with the other two control groups (Figure 5E), indicating that induction of B7‐H3 CAR‐T cells could promote the elimination of cancer cells by immune effects in the mice. In addition, the degree of intratumoral infiltration of CD3+ T cells and cancer cell apoptosis were found to increase, and the induced proliferative activity of tumor cells as probed by the marker Ki67 was decreased in the mice of groups four and five compared with the other three control groups (Figure 5F).

### 2.5 Combined Treatment of Cr@PD‐Alp with B7‐H3 CAR‐T Cells

Clinically, PIK3CA gene mutations occur frequently in malignant breast tumors, which are significantly correlated with resistance to chemotherapy and immunotherapy, tumor metastasis, and poor survival.^[^ ^22^ ^]^ We believed that Cr@PD‐Alp, which contains a Cr@PD core that can induce a photothermal effect for cell killing and an Alp‐encapsulated shell that can cause additional cytotoxicity through chemotherapy, could synergize CAR‐T cell therapy to combat tumors harboring these mutations. In fact, Alp is the first FDA‐approved PI3K inhibitor for therapy in patients with advanced estrogen receptor‐positive breast cancer harboring PIK3CA mutations and PIK3CA‐related overgrowth spectrum syndrome.^[^ ^23^ ^]^ Combined with other treatment modalities, it can induce a synergistic antitumor effect through inhibition of the PI3K/Akt/mTOR signaling pathway.^[^ ^24^ ^]^ It was expected that in the TME containing Cr(III) ions and CAR‐T cells, this drug would exhibit an enhanced therapeutic efficacy.

We first screened the cytotoxicity of Alp against a range of hepatocellular carcinoma and breast cancer cell lines (Figure S6A and S6B, Supporting Information, respectively) after incubation for 24 h. It was found that Alp was particularly potent toward T‐47D cells, which have high expression of PIK3CA with the H1047R mutation. The corresponding IC_50_ value was down to 0.1 µg mL^−1^. We then constructed MDA‐MB‐231 PIK3CA‐wildtype and PIK3CA (H1047R/+) cell lines and examined the cytotoxicity of Alp against these cells (Figure S6C, Supporting Information). Interestingly, the PIK3CA (H1047R/+) cells were found to be more susceptible to Alp than the wildtype counterpart. The IC_50_ values were determined to be 1.8 versus 11.4 µg mL^−1^, respectively. In addition, we also assessed whether Alp would inhibit the PI3K/Akt/mTOR pathway and its downstream molecular components leading to apoptosis, using Western blot analysis. The results showed that Alp selectively inhibited the expression of the pAkt and pS6 ribosomal proteins and caused compensatory upregulation of Ras/Raf/MAPK pathway signaling (Figure S7, Supporting Information).

**Figure 6:**
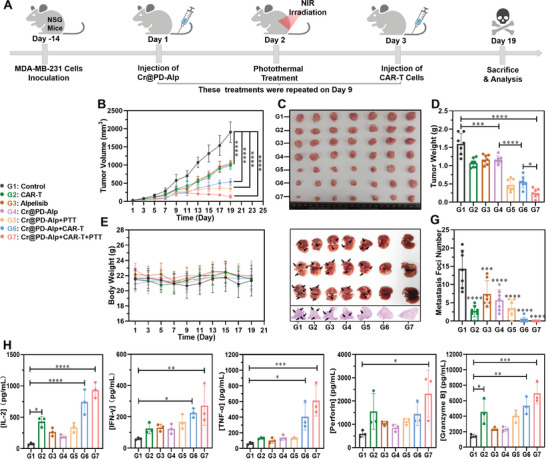
A) Timeline of the in vivo study of the synergistic antitumor effects of Cr@PD‐Alp, laser irradiation, and B7‐H3 CAR‐T cells against NSG mice bearing an MDA‐MB‐231 PIK3CA (H1047R/+) tumor. B) Tumor growth curves for the mice receiving different treatments: (G1) intravenous injection with PBS (100 µL); (G2) intravenous injection with B7‐H3 CAR‐T cells (3 × 10^6^, 100 µL); (G3) intravenous injection with Alp in PBS (8 mg kg^−1^, 100 µL); (G4) intravenous injection with Cr@PD‐Alp in PBS (10 mg kg^−1^, 100 µL); (G5) intravenous injection with Cr@PD‐Alp in PBS (10 mg kg^−1^, 100 µL) followed by laser irradiation (808 nm, 1 W cm^−2^, 8 min) at the tumor site after 24 h; (G6) intravenous injection with Cr@PD‐Alp in PBS (10 mg kg^−1^, 100 µL) followed by intravenous injection with B7‐H3 CAR‐T cells (3 × 10^6^, 100 µL) after 48 h; (G7) intravenous injection with Cr@PD‐Alp in PBS (10 mg kg^−1^, 100 µL) followed by laser irradiation (808 nm, 1 W cm^−2^, 8 min) at the tumor site after 24 h, and then intravenous injection with B7‐H3 CAR‐T cells (3 × 10^6^, 100 µL) after a further 24 h (n = 7). For all the groups, the same treatments were repeated on day 9. C) Photographs and D) weights of the tumors harvested from the different treatment groups of mice on day 19 (n = 7). E) Changes in the body weights of the mice in different groups (n = 7). F) Photographs and H&E stained images of the lungs showing the metastatic foci of the tumors (indicated with black arrows) on day 19 after different treatments. G) Statistical analysis of the tumor metastasis foci in the lungs (n = 7). H) Quantitative analysis of IL‐2, IFN‐γ, TNF‐α, perforin, and granzyme B for the mice in different groups on day 19 by ELISA (n = 3). For B), D), E), G), and H), data are expressed as the mean ± SD (n = 7 or 3). *p < 0.05, **p < 0.01, ***p < 0.001, and ****p < 0.0001.

After performing these in vitro studies, a human breast cancer model with PIK3CA mutations was established in NSG mice through the injection of MDA‐MB‐231 PIK3CA (H1047R/+) cells to study the combined therapeutic effects of Cr@PD‐Alp and B7‐H3 CAR‐T cells. When the tumors had grown to a size of 50–100 mm^3^ over a period of 14 days, the tumor‐bearing mice were randomly divided into seven groups (n = 7) and subjected to different treatments as described in **Figure** **6**. To cause a stronger therapeutic effect, particularly owing to the highly malignant nature of breast cancer cells, the sequential treatments of Cr@PD‐Alp, laser irradiation, and B7‐H3 CAR‐T cells were repeated on day 9 (Figure 6A). During the course of the treatments, the tumor volume and body weight of the mice were recorded every two days. At the end of the treatments on day 19, the mice in each group were sacrificed for further analysis. As shown in Figure 6B, the sole treatments of B7‐H3 CAR‐T cells, Alp, or Cr@PD‐Alp could induce a significant tumor inhibition effect. By combining the treatment of Cr@PD‐Alp and laser irradiation or Cr@PD‐Alp and B7‐H3 CAR‐T cells, the treatment efficacy could be further enhanced. Interestingly, combining all the three treatments (i.e., group seven) could achieve the highest efficacy. These results were consistent with the trends of the size and weight of the tumors harvested at the end of the treatments (Figure 6C,D). For all these treatment groups, the weight of the mice was not significantly changed over the treatment period (Figure 6E).

In addition, tumor metastasis to the lungs, which is a common phenomenon for advanced tumors, was also examined after the mice were sacrificed. As shown in Figure 6F,G, while several tumor metastasis loci (indicated with black arrows) were observed for all the control groups (no. 1–6), almost no metastasis was observed in the lungs for the treatment group seven, indicating that combining the therapeutic effects of Cr@PD‐Alp, laser irradiation, and B7‐H3 CAR‐T cells could induce photo‐metallo‐immunotherapy for effective prevention of tumor metastasis to the lungs.

At the end of the treatments on day 19, the levels of different intratumoral cytokines, including IL‐2, IFN‐γ, and TNF‐α, as well as the proteins perforin and granzyme B were measured via ELISA for all groups of mice. The results showed that the concentrations of all these immune markers were significantly elevated and attained the highest values for treatment group seven compared with the other control groups (Figure 6H). Finally, the tumors were harvested on day 19 for histological staining (Figure S8, Supporting Information). For treatment group seven, the degree of intratumoral infiltration of CD3+ T cells was slightly higher than that for group six and was significantly higher than that for groups one and two. TUNEL assay showed that the extent of apoptosis was remarkable for groups five to seven, while that for the other control groups was significantly lower. The results were consistent with those obtained in the histological evaluation of Ki67 proliferative marker, in which the expression of Ki67 was also significantly lower for groups five to seven.

### 2.6 Cr^3+^‐Promoted T‐Cell Migration, Chemokine Secretion, and TLS Formation

**Figure 7:**
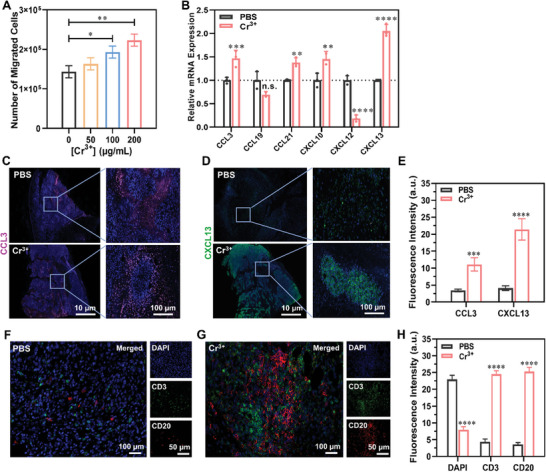
A) Effect of the concentration of Cr^3+^ ions on the migration of B7‐H3 CAR‐T cells. B) Real‐time fluorescence‐based quantitative PCR detection of chemokine gene expression in Hep3B cells after stimulation with PBS or Cr^3+^ ions (200 µg mL^−1^) for 24 h. Immunofluorescence staining of the chemokines C) CCL3 and D) CXCL13 in the tumor tissues of the mice being treated with PBS or CrCl_3_ (10 mg kg^−1^) in PBS (50 µL) and E) the corresponding relative fluorescence intensities. Formation of TLSs as indicated by the aggregates of CD3+ T cells (green) and juxtaposed CD20+ B cells (red) in the tumor tissues of the mice being treated with F) PBS or G) CrCl_3_ in PBS and H) the corresponding relative fluorescence intensities. For A), B), E), and H), data are expressed as the mean ± SD (n = 3). n.s., not significant, *p < 0.05, **p < 0.01, ***p < 0.001, and ****p < 0.0001.

According to our previous study,^[^ ^11^ ^]^ Cr NPs can promote the migration ability of macrophages and dendritic cells. In aqueous media and cell culture, they undergo degradation to release Cr^3+^ ions. Therefore, we further investigated the role of Cr^3+^ ions on B7‐H3 CAR‐T cells in the TME. First, we assessed the ability of Cr^3+^ ions to promote the migration of B7‐H3 CAR‐T cells. As shown in **Figure** **7**A, the number of migrated cells increased significantly with the concentration of Cr^3+^ ions. We then incubated Hep3B cells with Cr^3+^ ions and then detected the expression of chemokine genes in the cells by real‐time fluorescence‐based quantitative polymerase chain reaction (PCR). It was found that Cr^3+^ ions could significantly promote the mRNA expression of various chemokines, including CCL3, CCL21, CXCL10, and CXCL13, but not CCL19 and CXCL12 (Figure 7B). Similar results were obtained when MDA‐MB‐231 breast cancer cells were used (Figure S9, Supporting Information).

The study was then extended to a mouse model with a subcutaneous hepatocellular carcinoma induced by injecting H22 cells to BALB/c mice. Once the tumor volume reached ≈100 mm^3^, the mice were injected intratumorally with PBS or CrCl_3_ (10 mg kg^−1^) in PBS (50 µL) on Just 1 and day 7. The tumors were then removed from the mice on day 14 and subjected to immunofluorescence staining. It was found that the expression of the chemokine genes CCL3 and CXCL13 was significantly higher in the tumors of the Cr^3+^‐treated mice than those of the PBS‐treated control group (Figure 7C–E), which were consistent with the in vitro results (Figure 7B). However, for CXCL12, the expression was not found to be lower for the Cr^3+^‐treated group (Figure S10, Supporting Information) as observed in Hep3B cells.

Finally, we further examined the degree of infiltration of T cells and B cells in the tumor of the mice. Interestingly, we found a large number of CD3+ T cells and CD20+ B cells infiltrating the tumor in the Cr^3+^‐treated mice and that the CD3+ T‐cell aggregates (green) and juxtaposed CD20+ B‐cell aggregates (red) formed a TLS structure, which was not observed in the PBS‐treated control group (Figure 7F–H).

## 3 Conclusion

CAR‐T cell therapy is a mainstream cell‐based therapeutic method for cancers. However, the main drawbacks of existing CAR‐T cell treatments are the relatively weak cell‐killing efficiency and insufficient intratumoral infiltration, especially in the immunosuppressive TME. We report herein an extension of our previous study of photo‐metallo‐immunotherapy,^[^ ^11^ ^]^ using Cr NPs coated with a layer of polydopamine with or without entrapped Alp to promote CAR‐T cell migration and boost the therapeutic efficacy against solid tumors. Through a series of in vitro and in vivo experiments, including the use of NSG mice engrafted with a Hep3B liver tumor or an MDA‐MB‐231 PIK3CA‐mutated breast tumor as the animal models, we have demonstrated that these nanocomposites can induce mild hyperthermia upon laser irradiation (at 808 nm), and this effect can boost the therapeutic effect of B7‐H3 CAR‐T cells and prolong the survival of the animals through promoting the infiltration of the CAR‐T cells and the secretion of serum cytokines, including IL‐2, IFN‐γ, and TNF‐α. With Alp as an additional chemotherapeutic agent, Cr@PD‐Alp exhibits superior potency toward the NSG mice bearing an MDA‐MB‐231 PIK3CA (H1047R/+) tumor and can inhibit tumor metastasis to the lungs. Further studies have demonstrated that Cr^3+^ ions, which are the major degradation product of these nanocomposites, can promote the migration of B7‐H3 CAR‐T cells and the CCL3 and CKCL13 chemokine expression, facilitating the formation of TLS in the tumor tissues. The overall results show that these tailor‐made Cr‐based nanocomposites can empower the CAR‐T cell therapy against solid tumors.

## 4 Experimental Section

### Preliminary Treatment of Cr NPs

Cr particles were supplied by Suzhou Beike Nano Technology. With an initial diameter of 5–10 µm, these particles were dispersed in deionized water at a concentration of 1 mg mL^−1^. The dispersion was then sonicated using a probe at 0.46 kW h^−1^ in a water bath at ambient temperature for 24 h. At the end of the sonication, the dispersion was centrifuged at a speed of 12000 rpm for 20 min to collect the supernatant. The supernatant was subsequently filtered through a 0.1 µm filter membrane and then concentrated to yield Cr NPs at a concentration of 100 µg mL^−1^ for the following studies.

### Fabrication of Cr@PD

A dispersion of Cr NPs (100 µg mL^−1^) in deionized water (1 mL) was first centrifuged at 12000 rpm for 20 min. The precipitate obtained was then mixed with 1 mL of dopamine (50 µg mL^−1^) and 1 mL of the L‐DOPA dimer (50 µg mL^−1^) in 10 mM Tris HCl at pH 8.5. Further buffer was then added to make a total volume of 20 mL. The mixture was then sonicated in a water bath for 3 h at room temperature. The precipitate obtained after centrifugation at 12000 rpm for 20 min was mixed with deionized water and centrifuged again at 12000 rpm for 20 min, and these washing and centrifugation steps were repeated for three times. The resulting Cr@PD NPs were dispersed in deionized water at a concentration of 100 µg mL^−1^.

### Fabrication of Cr@PD‐Pc

A dispersion of Cr@PD NPs was first centrifuged at 12000 rpm for 10 min. The precipitate obtained was mixed with Pc (10 mM, 1 mL) in water with 0.5% Tween 20. The mixture was stirred at room temperature for 16 h. After centrifugation at 12000 rpm for 20 min, Cr@PD‐Pc NPs were obtained and dispersed in deionized water to give a concentration of 100 µg mL^−1^. As determined by electronic absorption spectroscopy, the concentration of Pc was found to be 3.5 µM, giving a loading efficiency of 35%.

### Fabrication of Cr@PD‐Alp

A dispersion of Cr NPs (100 µg mL^−1^) in deionized water (1 mL) was first centrifuged at 12000 rpm for 20 min. The precipitate obtained was then mixed with 1 mL of dopamine (50 µg mL^−1^) and 1 mL of the L‐DOPA dimer (50 µg mL^−1^) in Tris HCl at pH 8.5, as well as 1 mL of Alp (20 µg mL^−1^) in absolute EtOH. Further buffer was then added to make a total volume of 20 mL. The mixture was then sonicated in a water bath for 3 h at room temperature. The precipitate obtained after centrifugation at 12000 rpm for 20 min was mixed with deionized water and centrifuged again at 12000 rpm for 20 min, and these washing and centrifugation steps were repeated for three times. The resulting Cr@PD‐Alp NPs were dispersed in deionized water at a concentration of 100 µg mL^−1^. As determined by electronic absorption spectroscopy, the concentration of Alp was found to be 16.4 µg mL^−1^, giving a loading efficiency of 82%.

### Characterization of the Nanocomposites

The hydrodynamic diameters and zeta potentials of the Cr‐based NPs were measured using a DeltaMax Pro analyzer. The morphology and elemental composition were examined using a JEOL JSM7800‐F scanning electron microscope. TEM images were obtained on a FEI Tecnai G2 Spirit transmission electron microscope operated at 120 keV acceleration voltage. Electronic absorption spectra and steady‐state fluorescence spectra were recorded using a Cary 5G UV‐Vis‐NIR spectrophotometer and a Hitachi F‐7000 spectrofluorometer, respectively. An ULVAC PHI 5000 Versa Probe II instrument was used for XPS analysis of the surface of Cr@PD using Al Kα radiation (λ = 0.83 nm, hν = 1486.7 eV). The X‐ray source was operated at 23.5 W, and the data were analyzed using MultiPak Version 9.0 software. Raman spectra were acquired using an InVia Reflex confocal Raman microscope with a 532 nm argon ion laser serving as the excitation source. A thermal imager (HIKMICRO, HM‐TPH10‐3AUF) was used to detect the temperature change in the dispersions of nanocomposites in deionized water subjected to 808 nm (1 W cm^−2^) laser irradiation.

### Cell Lines and Culture Conditions

MDA‐MB‐231 (ATCC, no. HTB26), 4T1 (ATCC, no. CRL‐2539), and Hep3B (ATCC, no. ATCC‐HB‐8064) cells were maintained in RPMI 1640 medium (Thermo Fisher Scientific, no. 23400‐021) supplemented with fetal bovine serum (FBS) (10%; Thermo Fisher Scientific, no. 10270‐106) and penicillin–streptomycin solution (100 unit mL^−1^ and 100 mg mL^−1^, respectively). Huh7 (ATCC, no. CCL‐185), BT‐20 (ATCC, no. HTB‐22), T‐47D (ATCC, no. HTB‐133), and MCF‐7 (ATCC, no. HTB‐22) were maintained in DMEM (Thermo Fisher Scientific, no. 12100‐046) supplemented with FBS (10%) and penicillin–streptomycin solution (100 unit mL^−1^ and 100 mg mL^−1^, respectively). All the cells were grown at 37 °C in a humidified 5% CO_2_ atmosphere. The plasmids pLV3‐CMV‐PIK3CA (human)‐A3140G‐6 × His‐3 × FLAG‐CopGFP‐Puro and pLV3‐CMV‐PIK3CA (human)−6 × His‐3 × FLAG‐CopGFP‐Puro were purchased from Zhuhai Shutong Technology.

### Cellular Uptake of Cr@PD‐Pc

Approximately 2 × 10^5^ MDA‐MB‐231 cells per well were seeded on 12‐well glass‐bottom plates and incubated in culture medium overnight at 37 °C in a 5% CO_2_ atmosphere. The cells were then incubated with Cr@PD‐Pc NPs (10 or 20 µg mL^−1^) in culture medium for different durations from 2 to 24 h. The intracellular fluorescence intensity of Pc (λ_ex_ > 610 nm) was examined and quantified using a Leica TCS SP8 high‐speed confocal microscope.

### Study of Photocytotoxicity

Approximately 1 × 10^4^ MDA‐MB‐231, 4T1, and Hep3B cells per well were seeded on 96‐well plates. The cells were incubated in culture medium overnight at 37 °C under 5% CO_2_. The cells were then incubated with various concentrations of Cr@PD (up to 100 µg mL^−1^) in culture medium for 8 h. After being rinsed with PBS for three times, the cells were irradiated with an 808 nm laser operated at 1 W cm^−2^ for 10 min. The cytotoxicity under various conditions was determined using the CCK8 assay. The results were compared with those without the laser irradiation. For MDA‐MB‐231 cells, the cytotoxicity was also examined using a live/dead double staining protocol. After the drug incubation and laser irradiation as described above, the cells were incubated with calcein‐AM and PI in solutions (Dojindo Laboratories) for 30 min as per the manufacturer's instructions. Live cells (which exhibit green fluorescence) and dead cells (which exhibit red fluorescence) were distinguished using an Olympus IX71 inverted fluorescence microscope.

### Study of Cell Death Mechanism

Approximately 2 × 10^5^ MDA‐MB‐231 cells per well were seeded on a 6‐well plate and incubated in culture medium at 37 °C under 5% CO_2_ for 12 h. The cells were then incubated with Cr@PD (100 µg mL^−1^) in culture medium for 8 h with or without further laser irradiation (808 nm, 1 W cm^−2^) for 10 min. The cell death mechanism was then determined using an annexin V‐FITC apoptosis kit according to the manufacturer's instructions.

### Preparation of B7‐H3 CAR‐T Cells

All viral vector production practices adhered to the regulatory guidelines. The lentiviral vector supernatant for B7‐H3 CAR‐T cells was produced by transient transfection of 293T cells (Takara Bio) with the corresponding CAR plasmid and three packaging plasmids: pLP1, pLP2, and pLP/VSVG. The medium was replaced at 4 h post‐transfection. After 48 h, the cell supernatant was collected, filtered through a 0.45 µm filter, and treated with benzonase (Merck) for 16 h. The harvest mixture was then passed through a Mustang Q ion‐exchange capsule (Pall). The Mustang Q membrane was washed with 50 mM Tris HCl buffet at pH 8.0 supplemented with 750 mM NaCl(aq), and then eluted in fractions using 50 mM Tris HCl buffet at pH 8.0 supplemented with 1.5 M NaCl(aq) and diluted with phosphate buffer (pH 7.2). The eluate was further concentrated approximately tenfold using a 300 kDa TFF column. The final concentrate was formulated with 2% human serum albumin, filtered through a 0.22 µm filter, divided into aliquots in 2 mL cryotubes, quickly frozen on dry ice, and stored at −80 °C.

Peripheral blood mononuclear cells from healthy donors (Leide Biosciences) were enriched using CD3 magnetic beads (Miltenyi Biotec) and stimulated using anti‐CD3/CD28 beads (Dynabeads, Human T Activator CD3/CD28; Life Technologies) at a 1:3 (bead:T cell) ratio. The cells were then cultured in H3 medium (Takara Bio) supplemented with 4% human AB serum and 300 unit mL^−1^ recombinant human IL2 (T&L Biotechnology). The cells were exposed to lentivirus‐containing supernatant at a multiplicity of infection of 5 on day 2 and day 3. The beads were magnetically removed on day 4 or day 5, after which the cells were further expanded for 3–5 days in H3 medium supplemented with 300 unit mL^−1^ recombinant human IL2 before the in vitro and in vivo studies. The cells were harvested and cryopreserved.

### Cytotoxicity of B7‐H3 CAR‐T Cells

To evaluate the specific cell‐killing ability of B7‐H3 CAR‐T cells, the cells were first incorporated with a specific firefly luciferase‐based reporter gene system to target MDA‐MB‐231 and Hep3B cells, respectively. The CAR‐T cells were then cocultured with these cancer cells at different E:T ratios for 24 h. The absorbance at 600 nm was measured immediately after adding the substrate luciferin to each well to determine the cell viability. The results were comparted with those for Mock‐T cells.

To study the effect of Cr@PD, B7‐H3 CAR‐T cells and Hep3B cells at an E:T ratio of 1:1 were cocultured on 12‐well plates for 12 h. The cells were then incubated in culture medium with or without Cr@PD (200 µg mL^−1^) for 24 h and further laser irradiation (808 nm, 1 W cm^−2^) for 10 min. The mortality rate of Hep3B cells was determined by bioluminescence using an imaging system (Xenogen IVIS‐Spectrum). After incubating B7‐H3 CAR‐T cells with Cr@PD or Cr@PD‐Alp for 24 h, the cytokines IL‐2, IFN‐γ, and TNF‐α in the supernatant were measured using ELISA kits (Thermo Fisher Scientific).

To study the cytotoxicity of migrated B7‐H3 CAR‐T cells, Hep3B cells (5 × 10^5^ per well) were seeded in the lower chamber. After 12 h, B7‐H3 CAR‐T cells (1 × 10^6^ per well) were added to the upper chamber containing the culture medium. Cr@PD or Cr@PD‐Alp (200 µg mL^−1^) in fresh medium containing 10% FBS was then added to the lower chamber. The upper chamber was removed after 6 h, and the cell supernatant from the cells in the lower chamber was collected. The levels of perforin and granzyme B in the supernatant were measured using ELISA kits (Thermo Fisher Scientific).

### In Vivo Biodistribution of Cr@PD‐Pc

All animal experiments were approved by the Animal Experimentation Ethics Committee of Huazhong University of Science and Technology Union Shenzhen Hospital (Nanshan Hospital) (Ref. No. QA‐0007‐F01‐00). In situ mammary tumors were established in BALB/c nude mice using MDA‐MB‐231 cells. Once the tumor volume reached 100–200 mm^3^, the mice were divided into two groups. The mice in each group were intravenously injected with either PBS or Cr@PD‐Pc NPs (1 mg mL^−1^, n = 4) (200 µL). The biodistribution of Cr@PD‐Pc was assessed by detecting the fluorescence signals using an imaging system (Xenogen IVIS‐Spectrum) at 24 and 48 h post‐injection. Tumor tissues and major organs, including heart, liver, spleen, lung, and kidneys were collected from the mice at these time points, after which the fluorescence signals were detected.

### In Vivo Toxicity of Cr@PD

C57BL/6 female mice (6 weeks old, 16–20 g) were purchased from Shanghai Southern Model Biotechnology and housed under specific pathogen‐free (SPF) conditions. The in vivo toxicity of Cr@PD was determined as follows: C57BL/6 mice were randomly divided into two to three groups (n = 3 to 4), and each group was intravenously injected with either PBS or Cr@PD NPs (10 or 20 mg kg^−1^) in PBS (100 µL). Serum, blood, and major organs, including heart, liver, spleen, lung, kidneys, brain, and intestine were collected from the mice in different groups on days 1, 7, 30, and 90 after the injection. Liver and kidney function tests, routine blood tests, and H&E staining were performed to assess the toxicity.

### In Vivo Antitumor Efficacy of Cr@PD Combined with CAR‐T Cells

Female NSG mice (6‐7 weeks old, 16–20 g) were procured from Shanghai Southern Model Biotechnology. A subcutaneous model of human Hep3B hepatocellular carcinoma was established in these mice by injecting 5 × 10^6^ Hep3B cells subcutaneously into the right abdomen of each mouse. Once the tumor volume reached ≈200 mm^3^, the mice were randomly divided into five groups (n = 5): (G1) intravenous injection with PBS (100 µL); (G2) intravenous injection with Cr@PD in PBS (10 mg kg^−1^, 100 µL); (G3) intravenous injection with B7‐H3 CAR‐T cells (3 × 10^6^, 100 µL); (G4) intravenous injection with Cr@PD in PBS (10 mg kg^−1^, 100 µL) followed by intravenous injection with B7‐H3 CAR‐T cells (3 × 10^6^, 100 µL) after 48 h; (G5) intravenous injection with Cr@PD in PBS (10 mg kg^−1^, 100 µL) followed by laser irradiation (808 nm, 1 W cm^−2^, 8 min) at the tumor site after 24 h, and then intravenous injection with B7‐H3 CAR‐T cells (3 × 10^6^, 100 µL) after a further 24 h (n = 5). During laser irradiation, the mice were anesthetized with isoflurane. Tumor volume was measured every two days using a digital caliper, and the mice were euthanized when the tumor volume exceeded 2000 mm^3^. The survival time of the mice was recorded over a period of 30 days.

### Cytotoxicity of Alp

A range of hepatocellular carcinoma and breast cancer cell lines, including Huh7, Hep3B, MDA‐MB‐231, BT‐20, T‐47D, and MCF‐7, as well as MDA‐MB‐231 PIK3CA‐wildtype and PIK3CA (H1047R/+) cell lines were used in this study. The cells (1 × 10^4^ cells per well) were seeded on 96‐well plates, and then incubated with different concentrations of Alp (2–10 µg mL^−1^) in culture medium for 24 h. The cell viability was then assessed using a CCK8 assay, and the readings were taken using a Synergy HT multi‐detection enzymatic spectrometer (BioTek). The percentage of viable cells was then calculated.

### Western Blotting

Cultures were repeated three times on 6‐well plates at a density of 1 × 10^6^ cells per well. After treatment with Alp (2–10 µg mL^−1^) for 48 h, the cells were harvested and lysed in a radioimmunoprecipitation (RIPA) buffer containing a cocktail of proteases and phosphatase inhibitors (Beyotime). The lysates were then quantified using the BCA Protein Quantification Kit (Beyotime). Proteins containing 20 µg of total protein were separated by 10% SDS‒PAGE (Beyotime) and transferred to a polyvinylidene difluoride membrane (Millipore) by electroblotting. The membrane was blocked with 5% BSA for 1 h at room temperature and then incubated with primary antibodies overnight at 4 °C. The membrane was then washed thrice with a PBS solution and incubated with the corresponding horseradish peroxidase (HRP)‐conjugated immunoglobulins. Enhanced chemiluminescence reagents (Thermo Fisher Scientific) were used to analyze the blots using Image Lab (Bio‐Rad).

### In Vivo Antitumor Efficacy of Cr@PD‐Alp Combined with B7‐H3 CAR‐T Cells

Female SG mice (6‐7 weeks old, 16–20 g) were procured from Shanghai Southern Model Biotechnology and maintained under SPF conditions. A human MDA‐MB‐231 breast cancer model was established in NSG mice by subcutaneously injecting a 2:1 mixture of MDA‐MB‐231 PIK3CA‐WT cells and PIK3CA (H1047R/+) mutant cells (5 × 10^6^ cells in total) into the mammary gland of each mouse. Once the tumor volume reached 50–100 mm^3^, the mice were randomly divided into seven groups (n = 7): (G1) intravenous injection with PBS (100 µL); (G2) intravenous injection with B7‐H3 CAR‐T cells (3 × 10^6^, 100 µL); (G3) intravenous injection with Alp in PBS (8 mg kg^−1^, 100 µL); (G4) intravenous injection with Cr@PD‐Alp in PBS (10 mg kg^−1^, 100 µL); (G5) intravenous injection with Cr@PD‐Alp in PBS (10 mg kg^−1^, 100 µL) followed by laser irradiation (808 nm, 1 W cm^−2^, 8 min) at the tumor site after 24 h; (G6) intravenous injection with Cr@PD‐Alp in PBS (10 mg kg^−1^, 100 µL) followed by intravenous injection with B7‐H3 CAR‐T cells (3 × 10^6^, 100 µL) after 48 h; (G7) intravenous injection with Cr@PD‐Alp in PBS (10 mg kg^−1^, 100 µL) followed by laser irradiation (808 nm, 1 W cm^−2^, 8 min) at the tumor site after 24 h, and then intravenous injection with B7‐H3 CAR‐T cells (3 × 10^6^, 100 µL) after a further 24 h. For all the groups, the same treatments were repeated on day 9. During laser irradiation, the mice were anesthetized with isoflurane. Tumor volume and body weight of the mice were measured every two days using a digital caliper.

### Immunohistochemical and Immunofluorescence Staining, TUNEL Assay, and Cytokine Detection

For immunohistochemical staining, the sections were incubated with an anti‐Ki67 antibody (Servicebio, no. GB111499, 1:1000) overnight at 4 °C, followed by incubation with 1× secondary antibody solution (50 µL) for 1 h (Servicebio, no. GB23001, 1:200). The sections were then stained with 3,3′‐diaminobenzidine and hematoxylin to detect the signal. For immunofluorescence staining, tumors were collected at 48 h after the B7‐H3 CAR‐T cell therapy. The tumor tissues were fixed with 10% neutral buffered formalin, embedded in paraffin wax, and sliced. The tumor tissue sections were then incubated with an anti‐CD3 antibody (Servicebio, no. GB12014, 1:200) overnight at 4 °C, followed by incubation with 1× secondary antibody solution (50 µL) for 1 h (Servicebio, no. GB21301, 1:300). Sections were observed under a fluorescence microscope and images were captured. For TUNEL assay, the TUNEL assay kit for tumor sections (Servicebio, cat. no. G1501, 1:200) was used according to the manufacturer's protocol. For cytokine assay: blood was collected from different groups of mice and centrifuged (8000 rpm, 10 min), after which the serum was collected. ELISA kits (Thermo Fisher Scientific) were used to measure the serum concentrations of IL‐2, IFN‐γ, and TNF‐α.

### Cell Migration Assay

CrCl_3_ was procured from Aladdin Reagents. In vitro CAR‐T cell migration was assessed using a Transwell system (6.5 mm diameter, 8.0 µm pore size; Corning). B7‐H3 CAR‐T cells (1 × 10^6^ per well) were added to the upper chamber containing the culture medium, while Cr^3+^ was diluted to different concentrations with fresh medium containing 10% FBS and added to the lower chamber. After 6 h, the upper chamber was removed, and the cells in the lower chamber were collected and counted using a Nikon ECLIPSE Ti2 microscope.

### Cr^3+^‐Promoted Chemokine Secretion and TLS Formation

C57BL/6 female mice (6 weeks old, 16–20 g) were procured from Shanghai Southern Model Biotechnology and housed under SPF conditions. To establish a subcutaneous model of H22 hepatocellular carcinoma, 5 × 10^6^ H22 cells were injected subcutaneously into the right hip of each mouse. Once the tumor volume reached ≈100 mm^3^, the mice were randomly divided into two groups (n = 3): (G1) intravenous injection with PBS (50 µL); (G2) intravenous injection with CrCl_3_ in PBS (10 mg kg^−1^, 50 µL). The tumor volume was measured every two days using a digital caliper, and the mice were euthanized on day 14. The tumors were collected, fixed, and preserved according to standard procedures. The tumor sections were labeled with primary antibodies: CXCL13 (Proteintech, no. 10927‐1‐AP, 1:200), CXCL12 (Proteintech, no. 17402‐1‐AP, 1:200), and CCL3 (Thermo Fisher Scientific, no. MA5‐24364, 1:100).

### Statistical Analysis

Statistical analysis was performed using GraphPad Prism v.8.0 software. The results were analyzed using one‐way or two‐way ANOVA or Tukey's post hoc test for multiple comparisons. Differences in survival data were calculated using the Kaplan‒Meier method, and P values were determined using the log‐rank test. The results are expressed as the mean ± SD. *P* values less than 0.05 were considered as statistical significance.

## Conflict of Interest

The authors declare no conflict of interest.

## Author Contributions

Q.Z., K.L., and G.L. performed most of the experiments, analyzed the data, and wrote the manuscript. D.L., D.K.P.N., and Q.L. supervised and directed the research and reviewed and edited the manuscript. X.H., Y.Z., G.Y., M.L., and Y.S. helped perform some of the in vitro and in vivo experiments. E.Y.X., C.L., and S.W. assisted in the characterization of the NPs and some of the in vitro studies. All authors helped to improve the manuscript.

## Supporting information

Supporting Information

## Data Availability

The data that support the findings of this study are available in the supplementary material of this article.
