# Supplementary material for: Photo‐metallo‐immunotherapy: Fabricating Chromium‐Based Nanocomposites to Enhance CAR‐T Cell Infiltration and Cytotoxicity against Solid Tumors
Source: Adv Mater. 2024 Jul 1;37(2):2407425. doi: 10.1002/adma.202407425 (PMC11733712; doi:10.1002/adma.202407425)
Supplement: Supplementary file 1 — Supporting Information [file ADMA-37-2407425-s001.pdf]

ADVANCED MATERIALS

Supporting Information

for *Adv. Mater.*, DOI 10.1002/adma.202407425

Photo-metallo-immunotherapy: Fabricating Chromium-Based Nanocomposites to Enhance CAR-T Cell Infiltration and Cytotoxicity against Solid Tumors

Qingshuang Zou, Ke Liao, Guangchao Li, Xin Huang, Yongwei Zheng, Gun Yang, Min Luo, Evelyn Y. Xue, Chuanqing Lan, Shuai Wang, Yao Shen, Dixian Luo, Dennis K. P. Ng* and Quan Liu**

Supporting Information

Photo-metallo-immunotherapy: Fabricating Chromium-Based Nanocomposites to Enhance CAR-T Cell Infiltration and Cytotoxicity against Solid Tumors

Qingshuang Zou,[†] Ke Liao,[†] Guangchao Li,[†] Xin Huang, Yongwei Zheng, Gun Yang, Min Luo, Evelyn Y. Xue, Chuanqing Lan, Shuai Wang, Yao Shen, Dixian Luo, Dennis K. P. Ng,* and Quan Liu**

Q. Zou, K. Liao, X. Huang, G. Yang, Y. Shen, D. Luo, Q. Liu

Department of Laboratory Medicine, Huazhong University of Science and Technology Union Shenzhen Hospital (Nanshan Hospital), Shenzhen University, Shenzhen 518052, China

E-mail: luodixian_2@163.com

liu_quan2020@163.com

Q. Zou, E. Y. Xue, C. Lan, S. Wang, D. K. P. Ng

Department of Chemistry, The Chinese University of Hong Kong, Shatin, N.T., Hong Kong, China

E-mail: dkpn@cuhk.edu.hk

K. Liao

Institute of Pharmacy and Pharmacology, School of Pharmaceutical Science, Hengyang Medical School, University of South China, Hengyang, Hunan 421001, China

G. Li

Department of Hematology, The Affiliated Guangdong Second Provincial General Hospital of Jinan University, Guangzhou 510317, China

Y. Zheng, M. Luo

Research and Development Department, Guangzhou Bio-Gene Technology Co. Ltd., Guangzhou 510530, China

† These authors contributed equally to this work.

Part of a Special Collection for The Chinese University of Hong Kong 60th Anniversary

Contents

Table S1 Fabrication of Cr@PD using different ratios of Cr NPs, dopamine, and the L-DOPA dimer.

Figure S1 A) Photothermal effect of Cr NPs in deionized water upon irradiation with a 808 nm laser operated at 1 W cm^{-2}. The corresponding effect of Cr@PD (Figure 2I) is given again in B) for comparison.

Figure S2 A) Fluorescence images of the mice being intravenously injected with PBS or Cr@PD-Pc in PBS (1 mg mL^{-1}) (200 μL) at 24 and 48 h post-injection. B) Fluorescence intensities (per unit area) of the tumor and some main organs at 24 and 48 h post-injection of Cr@PD-Pc in PBS (1 mg mL^{-1}, 200 μL).

Figure S3 C57BL/6 mice were randomly divided into 2 groups (with 3 mice in each group) and treated with an intravenous injection of PBS or Cr@PD in PBS (10 mg kg^{-1}) (100 μL). Blood samples were collected on day 1, 7, and 30 and then subjected to analysis of the standard blood biochemical indexes for liver and kidney functions, including the total protein (TP), albumin (ALB), aspartate aminotransferase (AST), alanine aminotransferase (ALT), creatine (CRE), urea (Urea-2618), and uric acid (UA).

Figure S4 Pathological toxicity analysis by H&E staining of tissue sections from major organs (heart, liver, spleen, lung, kidney, brain, and intestine) harvested on day 1, 7, and 30 after intravenous injection of PBS or Cr@PD in PBS (10 mg kg^{-1}) (100 μL).

Figure S5 A-L) Complete blood count and biochemical tests of the liver and kidney functions. C57BL/6 mice were randomly divided into 3 groups (with 4 mice in each group) and treated with an intravenous injection of PBS or Cr@PD in PBS [10 mg kg^{-1} (low-dose, L) or 20 mg kg^{-1} (high-dose, H)] (100 μL). Blood

samples were collected on day 90 and then subjected to analysis of the standard blood tests, including white blood cell count (WBC), red blood cell count (RBC), hemoglobin (HB), hematocrit (HCT), mean corpuscular hemoglobin (MCH), platelet count (PLT), total protein (TP), aspartate aminotransferase (AST), alanine aminotransferase (ALT), creatine (CRE), uric acid (UA), and blood urea nitrogen (BUN). Data are expressed as the mean ± SD (n = 4). M) Pathological toxicity analysis by H&E staining of tissue sections from major organs (heart, liver, spleen, lung, kidney, brain, and intestine) harvested on day 90 after intravenous injection of PBS or Cr@PD in PBS [10 mg kg^{-1} (low-dose, L) or 20 mg kg^{-1} (high-dose, H)] (100 μL).

Figure S6 Cytotoxicity of Alp against a range of A) hepatocellular carcinoma and B) breast cancer cell lines as well as C) MDA-MB-231 PIK3CA-wildtype and PIK3CA (H1047R/+) cells. Data are expressed as the mean ± SEM of three independent experiments, each performed in quadruplicate. D) Corresponding IC_{50} values.

Figure S7 Western blotting analysis of PI3K/AKT/mTOR pathway and its downstream proteins in MDA-MB-231 cells being treated with different concentrations of Alp.

Figure S8 A) Immunohistochemical staining for CD3, TUNEL staining, and immunohistochemical staining for Ki67 in tumors after different treatments as defined in Figure 6.

Figure S9 Real-time fluorescence-based quantitative PCR detection of chemokine gene expression in MDA-MB-231 cells after stimulation with PBS or Cr^{3+} ions (200 μg mL^{-1}) for 24 h.

Figure S10 Immunofluorescence staining of the chemokine CXCL12 in tumor tissues of the mice being treated with PBS or $CrCl_3$ (10 mg kg^{-1}) in PBS (50 μL).

Table S1. Fabrication of Cr@PD using different ratios of Cr NPs, dopamine, and the L-DOPA dimer.[a]

Sample	Volume (μL)				Weight Ratio of Cr:Dopamine:L-DOPA Dimer	Hydrodynamic Diameter[b]
	Cr NPs (50 μg mL^{-1})	Dopamine (100 μg mL^{-1})	L-DOPA Dimer (100 μg mL^{-1})	Tris HCl (pH 8.5)		
1	100	0	0	900	1:0:0	68.2 ± 4.2
2	100	50	0	850	1:1:0	139.7 ± 18.8
3	100	100	0	800	1:2:0	289.2 ± 35.8
4	100	0	50	850	1:0:1	93.2 ± 19.9
5	100	25	25	850	1:0.5:0.5	83.5 ± 18.3
6	100	25	50	825	1:0.5:1	370.3 ± 11.3
7	100	50	50	800	1:1:1	148.3 ± 8.1
8	100	100	50	750	1:2:1	185.3 ± 15.9

[a] Solutions of Cr NPs, dopamine, and the L-DOPA dimer in Tris HCl at pH 8.5 were mixed in the same medium to give a 1000 μL mixture that was subjected to ultrasound irradiation at ambient temperature for 3 h. [b] Determined by DLS.

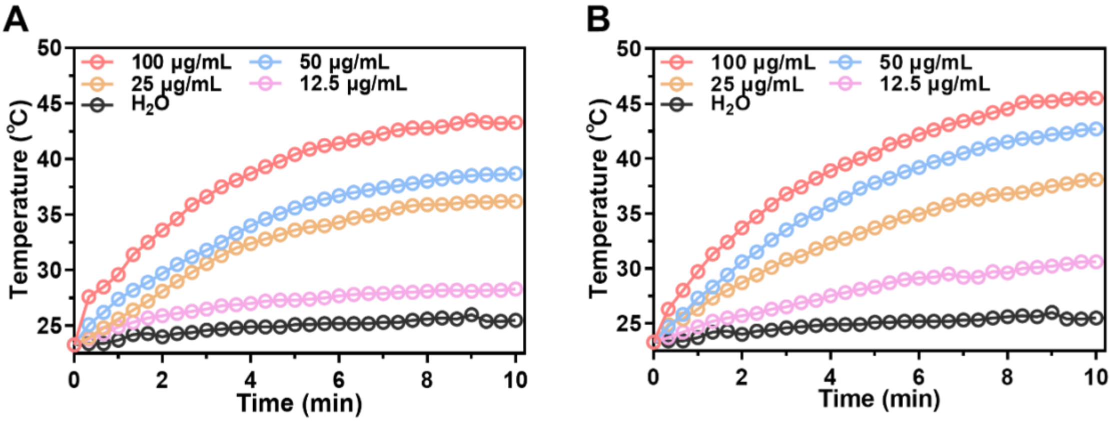

Figure S1. A) Photothermal effect of Cr NPs in deionized water upon irradiation with a 808 nm laser operated at 1 W cm^{-2}. The corresponding effect of Cr@PD (Figure 2I) is given again in B) for comparison.

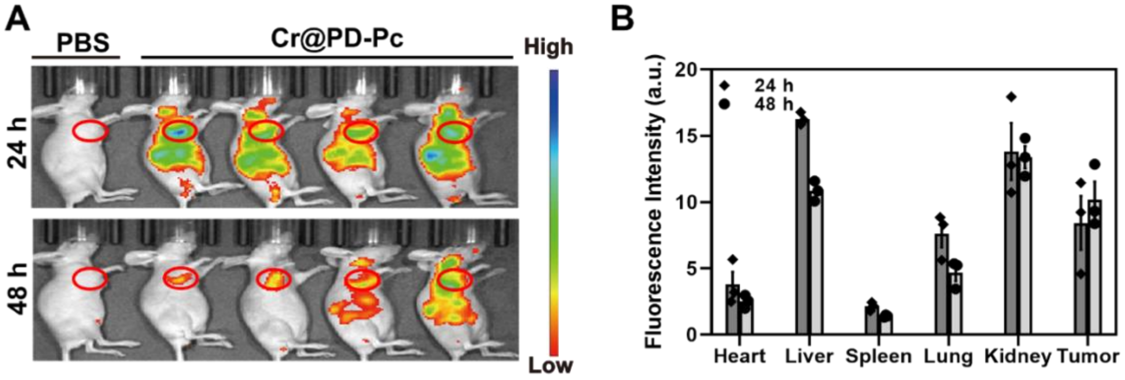

Figure S2. A) Fluorescence images of the mice being intravenously injected with PBS or Cr@PD-Pc in PBS (1 mg mL^{-1}) (200 μL) at 24 and 48 h post-injection. B) Fluorescence intensities (per unit area) of the tumor and some main organs at 24 and 48 h post-injection of Cr@PD-Pc in PBS (1 mg mL^{-1}, 200 μL). Data are expressed as the mean ± SD (n = 4).

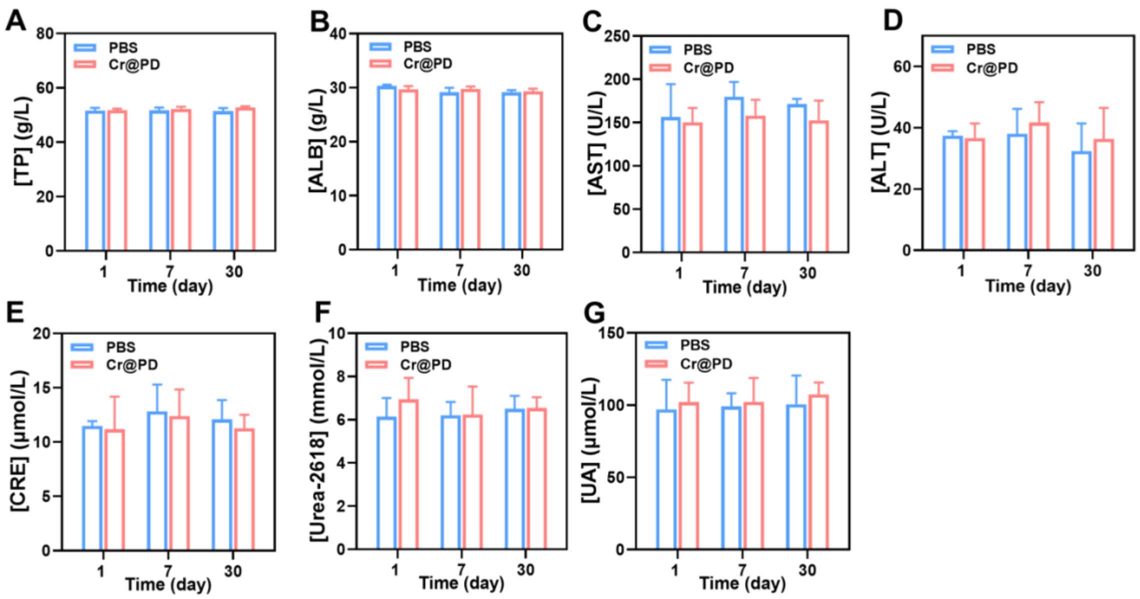

Figure S3. C57BL/6 mice were randomly divided into 2 groups (with 3 mice in each group) and treated with an intravenous injection of PBS or Cr@PD in PBS (10 mg kg^{-1}) (100 μL). Blood samples were collected on day 1, 7, and 30 and then subjected to analysis of the standard blood biochemical indexes for liver and kidney functions, including the total protein (TP), albumin (ALB), aspartate aminotransferase (AST), alanine aminotransferase (ALT), creatine (CRE), urea (Urea-2618), and uric acid (UA). Data are expressed as the mean ± SD (n = 3).

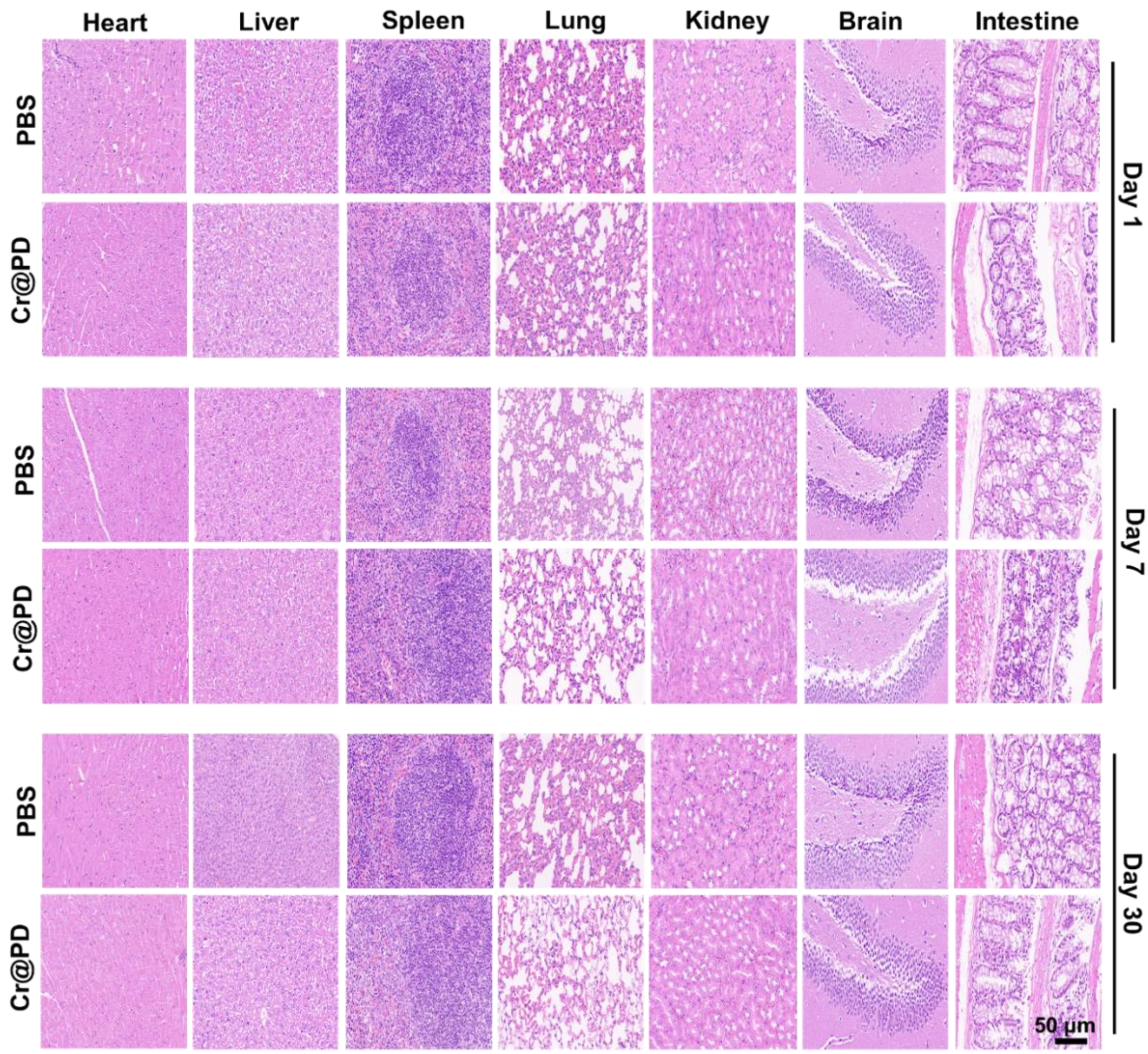

Figure S4. Pathological toxicity analysis by H&E staining of tissue sections from major organs (heart, liver, spleen, lung, kidney, brain, and intestine) harvested on day 1, 7, and 30 after intravenous injection of PBS or Cr@PD in PBS (10 mg kg^{-1}) (100 μL). Scale bars = 50 μm.

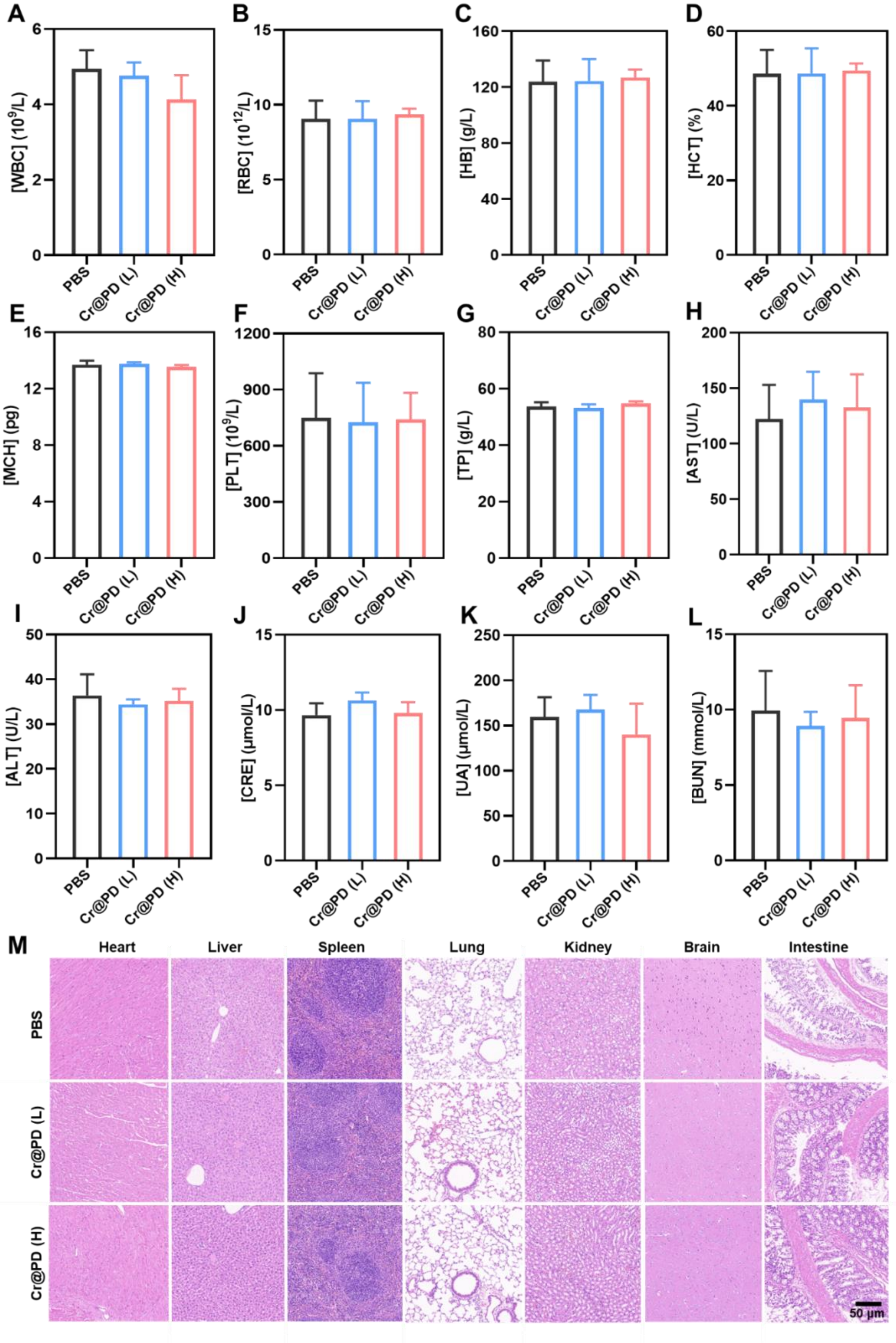
A
B
C
D
E
F
G
H
I
J
K
L
M
[WBC] (10^9/L)
[RBC] (10^12/L)
[HB] (g/L)
[HCT] (%)
[MCH] (pg)
[PLT] (10^9/L)
[TP] (g/L)
[AST] (U/L)
[ALT] (U/L)
[CRE] (μmol/L)
[UA] (μmol/L)
[BUN] (mmol/L)
PBS
Cr@PD (L)
Cr@PD (H)
Heart
Liver
Spleen
Lung
Kidney
Brain
Intestine
50 μm

Figure S5. A-L) Complete blood count and biochemical tests of the liver and kidney functions. C57BL/6 mice were randomly divided into 3 groups (with 4 mice in each group) and treated with an intravenous injection of PBS or Cr@PD in PBS [10 mg kg^{-1} (low-dose, L) or 20 mg kg^{-1} (high-dose, H)] (100 μL). Blood samples were collected on day 90 and then subjected to analysis of the standard blood tests, including white blood cell count (WBC), red blood cell count (RBC), hemoglobin (HB), hematocrit (HCT), mean corpuscular hemoglobin (MCH), platelet count (PLT), total protein (TP), aspartate aminotransferase (AST), alanine aminotransferase (ALT), creatine (CRE), uric acid (UA), and blood urea nitrogen (BUN). Data are expressed as the mean ± SD (n = 4). M) Pathological toxicity analysis by H&E staining of tissue sections from major organs (heart, liver, spleen, lung, kidney, brain, and intestine) harvested on day 90 after intravenous injection of PBS or Cr@PD in PBS [10 mg kg^{-1} (low-dose, L) or 20 mg kg^{-1} (high-dose, H)] (100 μL). Scale bars = 50 μm.

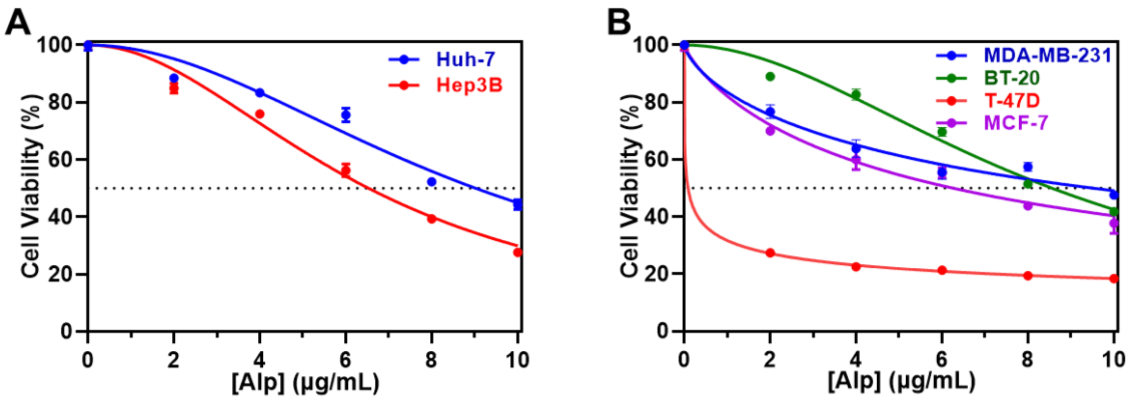

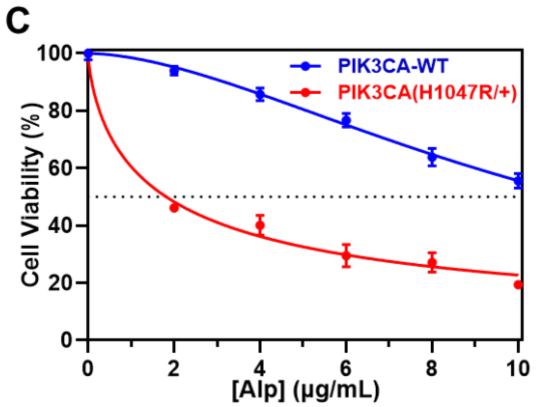

D

Cell Line	IC_{50} Value (μg mL^{-1})
Huh-7	9.0
Hep3B	6.5
MDA-MB-231	9.5
BT-20	8.6
T-47D	0.1
MCF-7	6.3
PIK3CA-WT	11.4
PIK3CA (H1047R/+)	1.8

Figure S6. Cytotoxicity of Alp against a range of A) hepatocellular carcinoma and B) breast cancer cell lines as well as C) MDA-MB-231 PIK3CA-wildtype and PIK3CA (H1047R/+) cells. Data are expressed as the mean ± SEM of three independent experiments, each performed in quadruplicate. D) Corresponding IC_{50} values.

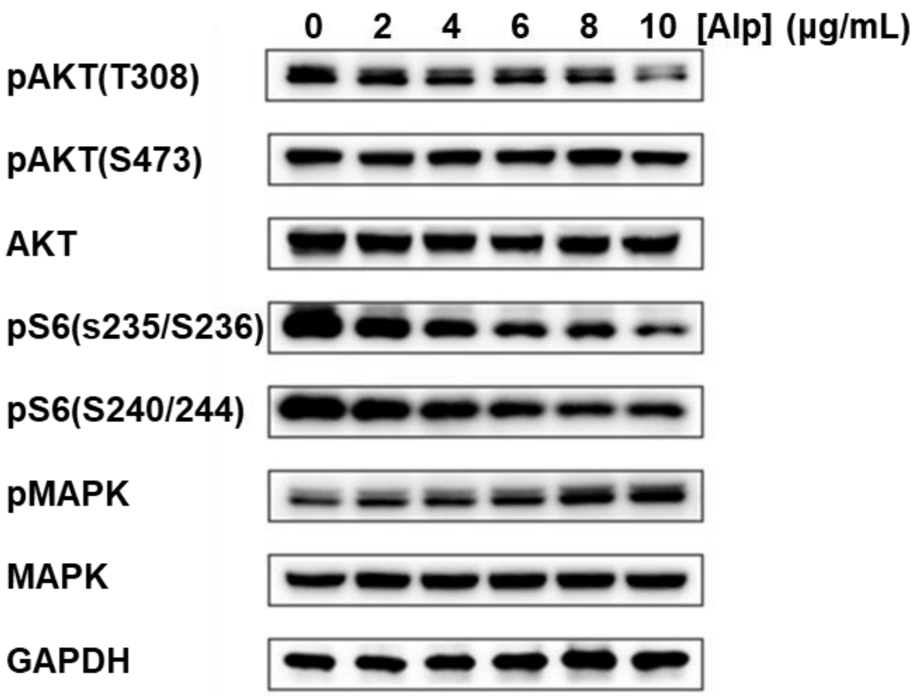

Figure S7. Western blotting analysis of PI3K/AKT/mTOR pathway and its downstream proteins in MDA-MB-231 cells being treated with different concentrations of Alp.

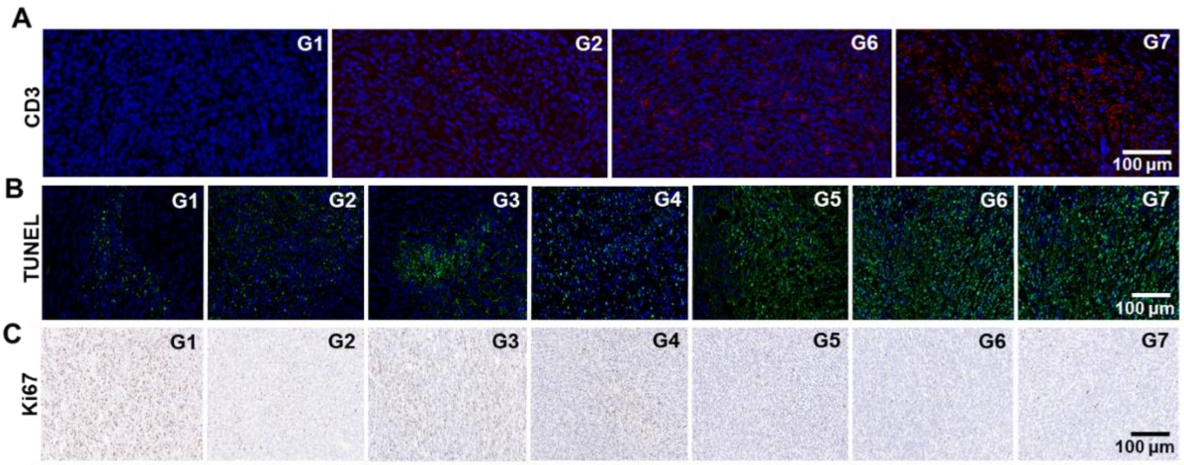

Figure S8. A) Immunohistochemical staining for CD3, TUNEL staining, and immunohistochemical staining for Ki67 in tumors after different treatments as defined in Figure 6. Scale bar = 100 µm.

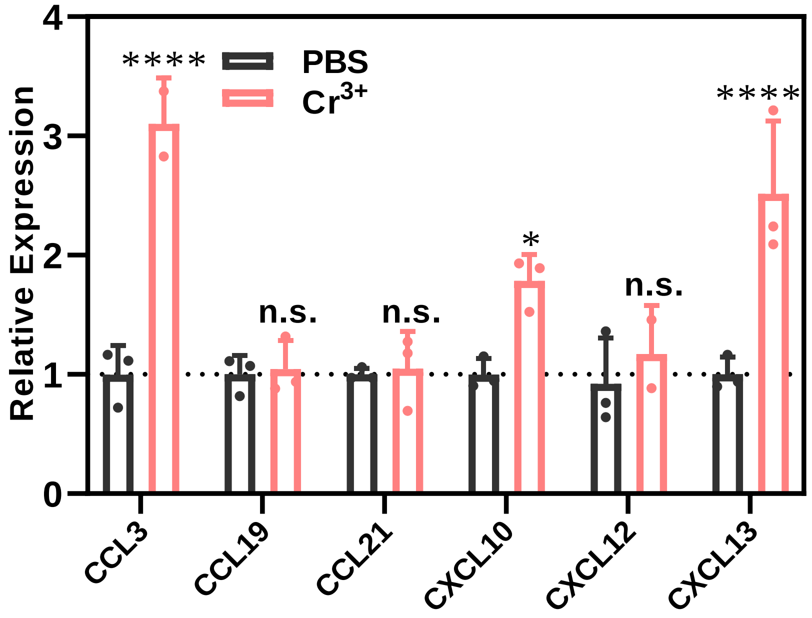

Figure S9. Real-time fluorescence-based quantitative PCR detection of chemokine gene expression in MDA-MB-231 cells after stimulation with PBS or Cr^{3+} ions (200 μg mL^{-1}) for 24 h. Data are expressed as the mean ± SD (n = 3). n.s., not significant, *$p < 0.05$, and ****$p < 0.0001$.

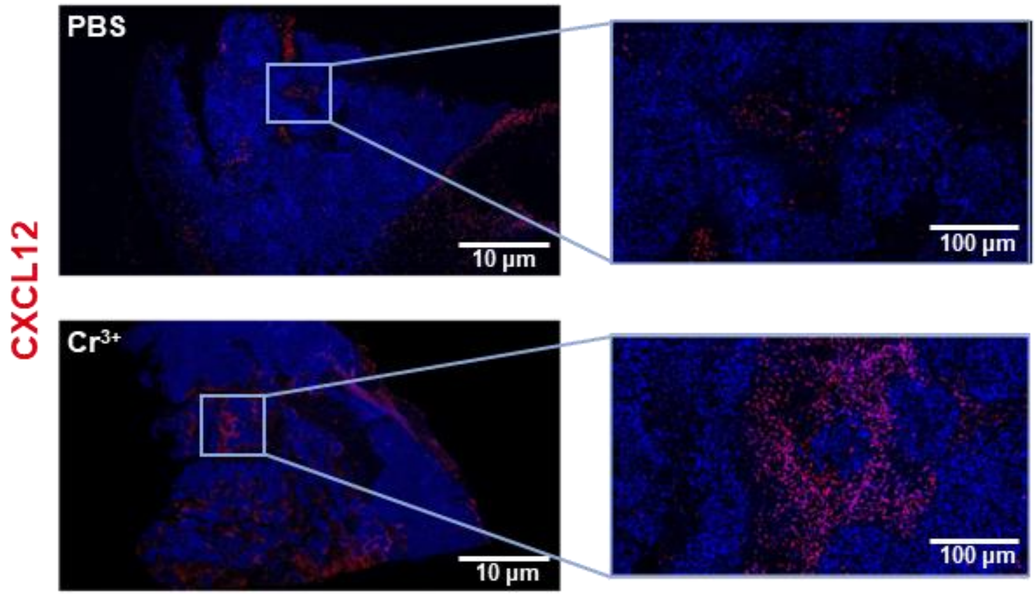

Figure S10. Immunofluorescence staining of the chemokine CXCL12 in tumor tissues of the mice being treated with PBS or $CrCl_3$ (10 mg kg^{-1}) in PBS (50 μL).
